# Gut-protective metabolic phenotype for diarrhoeal remission caused by an environmental probiotic thermophile

**DOI:** 10.1186/s40168-026-02476-9

**Published:** 2026-08-05

**Authors:** Hirokuni Miyamoto, Hideyuki Takahashi, Wataru Suda, Haruki Yamano, Yudai Inabu, Hiroaki Kodama, Yumiko Nakanishi, Shigeharu Moriya, Takashi Satoh, Tamotsu Kato, Chie Shindo, Naoko Tsuji, Makiko Matsuura, Chitose Ishii, Teruno Nakaguma, Tetsuji Etoh, Yuji Shiotsuka, Motoaki Udagawa, Atsushi Kurotani, Kenta Suzuki, Hiroshi Masuya, Satoshi Wada, Shinji Fukuda, Yukihiro Tashiro, Hisashi Miyamoto, Jun Kikuchi, Masahira Hattori, Takumi Nishiuchi, Naoki Yamamoto, Hiroshi Ohno

**Affiliations:** 1 RIKEN Center for Integrative Medical Sciences, Yokohama, Kanagawa 230-0045 Japan; 2 Graduate School of Horticulture, Chiba University, Matsudo, Chiba 271-8501 Japan; 3 Japan Eco-Science (Nikkan Kagaku) Co., Ltd., Chiba, Chiba 263-8522 Japan; 4 Sermas Co., Ltd, Ichikawa, Chiba 272-0033 Japan; 5 Graduate School of Medical Life Science, Yokohama City University, Yokohama, Kanagawa 230-0045 Japan; 6 Kuju Agricultural Research Center, Kyushu University, Takeda, Oita 878-0201 Japan; 7 Graduate School of Integrated Sciences for Life, Hiroshima University, Higashihiroshima, Hiroshima 739-8528 Japan; 8 RIKEN Center for Advanced Photonics, Wako, Saitama 351-0198 Japan; 9 Division of Hematology, Kitasato University School of Allied Health Sciences, Sagamihara, Kanagawa 252-0373 Japan; 10 Keiyo Gas Energy Solution Co. Ltd, Ichikawa, Chiba 272-0033 Japan; 11 Research Center for Agricultural Information Technology, National Agriculture and Food Research Organization, Tsukuba, Ibaraki 305-0856 Japan; 12 RIKEN, BioResource Research Center, Tsukuba, Ibaraki 305-0074 Japan; 13 Institute for Advanced Biosciences, Keio University, Tsuruoka, Yamagata 997-0052 Japan; 14 Gut Environmental Design Group, Kanagawa Institute of Industrial Science and Technology, Kawasaki, Kanagawa 210-0821 Japan; 15 Transborder Medical Research Center, Institute of Medicine, University of Tsukuba, Tsukuba, Ibaraki 305-8575 Japan; 16 Innovative Microbiome Therapy Research Center, Juntendo University Graduate School of Medicine, Tokyo, 113-8421 Japan; 17 Laboratory of Soil and Environmental Microbiology, Division of Systems Bioengineering, Department of Bioscience and Biotechnology, Faculty of Agriculture, Graduate School of Bioresources and Bioenvironmental Sciences, Kyushu University, Fukuoka, 819-0395 Japan; 18 Laboratory of Microbial Environmental Protection, Tropical Microbiology Unit, Center for International Education and Research of Agriculture, Faculty of Agriculture, Kyushu University, Fukuoka, 819-0395 Japan; 19 Miroku Co. Ltd, Kitsuki, Oita 873-0021 Japan; 20 RIKEN Center for Sustainable Resource Science, Yokohama, Kanagawa 230-0045 Japan; 21 School of Advanced Science and Engineering, Waseda University, Shinju-ku, Tokyo 169-8555 Japan; 22 Division of Integrated Omics Research, Research Center for Experimental Modeling of Human Disease, Bioscience Core Facility, Kanazawa University, Kanazawa, Ishikawa 920-8640 Japan; 23 Japan Institute for Health Security, Shinjuku-ku, Tokyo 162-8655 Japan

**Keywords:** Diarrhoeal remission, Compost, Caldifermentibacillus hisashii, Butyrate, 2-aminoisobutyric acid, Biosynthetic gene cluster, Gut microbiota, Livestock

## Abstract

**Background:**

Controlling diarrhoea in humans and livestock is a global challenge with diverse aetiologies. However, the dynamics of the microbiome for diarrhoeal remission are not sufficiently understood, and effective intervention strategies based on environmental microorganisms have not been fully explored.

**Methods:**

We investigated the metabolic structure associated with diarrhoeal remission using a combination of statistical, genomic, and proteomic approaches in a cattle model. Oral administration of the compost-derived thermophile *Caldifermentibacillus hisashii* significantly ameliorated persistent diarrhoea. Faecal bacterial populations and metabolites were characterised by a multi-step statistical pipeline comprising difference-in-differences (DID) analysis, Cliff's delta effect size estimation with permutation-based validation. The functional importance of the selected feature components was validated through genomic and proteomic analysis of *C. hisashii* N11 (AP028807.1).

**Results:**

Oral administration of *C. hisashii* significantly ameliorated persistent diarrhoea in calves. Although no significant differences in faecal bacterial community composition were observed, integrated analysis of faecal metabolites identified butyrate and 2-aminoisobutyrate (AIB) as the most discriminative features associated with diarrhoeal remission. Genomic and proteomic analyses of *C. hisashii* confirmed biosynthetic gene clusters for butyrate and AIB-containing lantibiotics, supporting the structural importance of these metabolites in diarrhoeal remission.

**Conclusion:**

These findings suggest that diarrhoeal remission observed in this study involves characteristic shifts in faecal metabolite profiles rather than marked changes in overall gut microbial community composition, highlighting a protective role of *C. hisashii* as an environmental probiotic against diarrhoeal dysbiosis through modulation of gut microbial metabolic output. This offers a perspective that bridges environmental microbiology and gut health within a One Health framework.

Video Abstract

**Supplementary Information:**

The online version contains supplementary material available at 10.1186/s40168-026-02476-9.

## Background

Diarrhoea is a global problem, causing 1.30 million deaths annually [1–3]. In addition, indigestion due to diarrhoea in livestock has a negative impact on productivity and increases mortality [3–5]. In general, diarrhoea is a clinical symptom of poor nutrient absorption owing to impaired water and electrolyte transport in the gastrointestinal tract; however, in many cases, identifying the cause of not only bacterial and viral infections but also other possible aetiologies is difficult. The intestinal tract of mammals is home to many diverse microorganisms, and a healthy gut microbiome is known to protect against damage to the epithelial cells of the intestinal tract. Therefore, prophylaxis with lactic acid bacteria as probiotics and faecal microbiota transplantation (FMT) have been attempted for the treatment of diarrhoea in recent years [6–9]. However, the composition and diversity of the gut microbial population are susceptible to various environmental factors that further affect human and animal health.

In recent years, attempts have been made to control the growth of plants and animals by using environmental microorganisms to control the symbiotic microbiota. In particular, research using members of the family Bacillaceae for this purpose is underway [10]. Our group reported the use of the thermophile Bacillaceae derived from compost to regulate the microbiota of soil and terrestrial plants [11, 12], marine sediments, marine plants (seagrass, a main target of blue carbon) [13], insects [14, 15], fish [16–18], livestock [19–22], and rodents [23, 24]. To date, compost-type fermented feeds containing thermophiles have been shown to contribute to the productivity of insects, fish, chickens, and pigs. Furthermore, as a method to reduce the burden on the environment, the effect of seagrass growth has been confirmed in the inshore near a fishery farm fed fermented feed containing thermophiles, suggesting that the symbiotic bacteria in the bottom sediments of seagrass changed after treatment. In addition, *Caldifermentibacillus hisashii* (synonym: *Caldibacillus hisashii* and *Bacillus hisashii*), a major functional thermophilic bacterium, was present in the fermented feed. *C. hisashii*, a member of the family Bacillaceae, is a functional thermophile candidate that can mimic the function of compost and was previously isolated from an experimental system consisting of germ-free mice implanted with compost [25, 26]. *C. hisashii* was first isolated as a species closely related to the *Bacillus thermoamylovorans* N11 strain [24]. It was originally registered as *B. hisashii* (type strain N-11^ T^ = NRBC 110226^ T^ = LMG 28201^ T^) [25] and was recently reclassified as *C. hisashii* [27]. Secretory IgA is a key component of mucosal immune defence in the gastrointestinal tract [28], and its deficiency has been associated with increased susceptibility to enteric infections and diarrhoea. Oral administration of *C. hisashii* to mice increased IgA levels. Administration to calves increases the abundance of the phylum Bacteroidetes (synonym: Bacteroidota) and reduces the abundance of the genus *Methanobrevibacter*, members of which are methane-producing bacteria, resulting in increased growth of calves [29]. These results suggest that the *Caldifermentibacillus* strain, a thermophilic environmental microorganism, is involved in the regulation of symbiotic microbial systems in various host species.

To evaluate the effects of thermophiles on diarrhoeal status, this study aimed to assess changes at the omics scale after the administration of the compost-derived thermophile *Caldifermentibacillus* as a feed additive. First, the conditions for diarrhoeal remission in cattle were observed. Interestingly, the short-term administration of the thermophile itself as a feed additive reduced the relative incidence of diarrhoea, although no significant changes were observed in the overall diversity of the gut bacterial community. However, the faecal metabolite groups common to the protective effects observed across individual conditions were statistically estimated via Cliff’s delta and permutation-based validation. Furthermore, genomic and proteomic analyses of *C. hisashii* were performed to validate the biosynthetic capacity underlying the identified feature metabolites. Thus, this study provides a novel perspective on the importance of symbiotic bacterial–metabolic interactions by examining the effects of an external, compost-derived thermophile on sustainable ecosystem conservation.

## Results

### Evaluation of the diarrhoea remission process

**Fig. 1:**
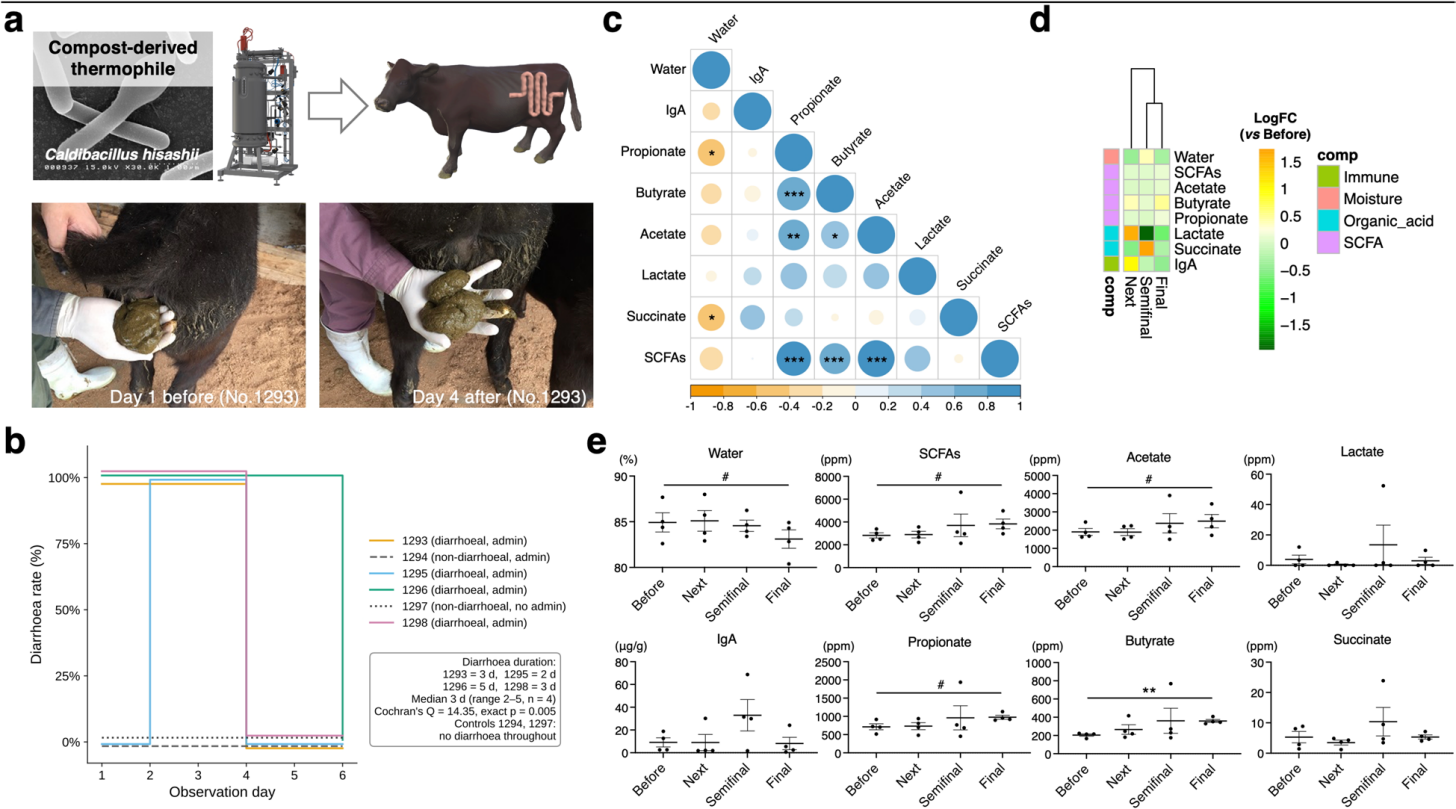
Evaluation of the process for controlling diarrhoea development. **a** Remission of diarrhoea in cattle due to the administration of environmental microorganisms. Photograph of the faeces of calf No. 1293 at the time of onset of diarrhoea (day 1 before) and 3 days after administration of the compost-derived thermophile *Caldifermentibacillus hisashii* (day 4 after, Final). **b** Individual diarrhoea trajectories of the four diarrhoeic calves (Nos. 1293, 1295, 1296, and 1298) and two non-diarrhoeic references (Nos. 1294 and 1297) over the observation period. The observation period was 4 days for Nos. 1293, 1295, and 1298, and 6 days for No. 1296. The temporal pattern of diarrhoea was evaluated using Cochran’s *Q *test with exact permutation (exact *p* = 0.005; 36,000 total permutations). **c** Spearman's rank correlation heatmap for faecal water, IgA, SCFA, lactate, and succinate levels. The colour scale represents the Spearman's rank correlation coefficient. **d** Fold changes in faecal water, IgA, SCFA, lactate, and succinate levels during diarrhoea remission. The LogFC values against “Before” (day 0 in Supplementary Fig. S1) were visualised. “Next”, “Semifinal”, and “Final” indicate day 1, day 2 (day 4 in No. 1296), and day 3 (day 5 in No. 1296), respectively, as described in Supplementary Fig. S1a. SCFA levels are the sum of acetate, butyrate, and propionate levels. **e** Changes in faecal water, IgA, SCFA, lactate, and succinate levels over time during the diarrhoea remission process. The terms “Before”, “Next”, “Semifinal”, and “Final” in Supplementary Fig. S1a are defined as described above. The asterisks indicate as follows: *, *p* < 0.05; **, *p* < 0.01; and ***, *p* < 0.001

Six calves were individually monitored in this study: four exhibited diarrhoea (Nos. 1293, 1295, 1296, and 1298) and two did not (Nos. 1294 and 1297). Five of the six calves received oral administration of *C. hisashii* for 4 days, with the exception of No. 1296, which received administration for 6 days. Calf No. 1297 did not receive the thermophile and served as an untreated reference. Calf No. 1294, which did not exhibit diarrhoea but received the thermophile, served as a non-diarrhoeal administered reference. The effects of administering the compost-derived thermophile *C. hisashii* to cattle were examined (Fig. 1a). Because a dam usually gives birth to only one calf, which is valuable and expensive, it is impossible to set up multiple test systems. Therefore, we analysed the faecal conditions of the six calves. In young cattle with diarrhoea (Supplementary Fig. S1a), a tendency towards recovery after *C. hisashii* administration was observed. In addition, as shown in Supplementary Figs. 1a and Supplementary S1a, the administration of the compost-derived thermophile to cattle resolved diarrhoea of unknown origin in a short period. The remission status of each individual in the study tended to improve after 3–5 days of treatment (Supplementary Figs. S1a and S1b).

The exact *p*-value was calculated as the proportion of permuted *Q* statistics equal to or exceeding the observed *Q*, without relying on the asymptotic chi-squared approximation, which may be inaccurate at small sample sizes. The temporal pattern of diarrhoea occurrence across the four diarrhoeic calves was statistically evaluated using Cochran’s *Q *test with exact permutation (exact *p* = 0.005; Fig. 1b), indicating that the reduction in diarrhoea incidence over the observation period was unlikely to have occurred by chance.

In addition, weakly expressed virulence genes that could not be detected by bacterial community analysis were investigated. An evaluation of the presence of pathogenic genes of *Escherichia coli* and *Salmonella* revealed that the tested calves were positive for the Shiga toxin gene (Nos. 1294, 1293, and 1295) (Supplementary Fig. S1c). The detected pathogenic genes from *E. coli* or *Salmonella* sp. were not always associated with diarrhoeal occurrence; therefore, the cause of the occurrence was unclear (Supplementary Fig. S1c). The faecal water, IgA, short-chain fatty acid (SCFA), lactate, and succinate levels in individual cattle were investigated over time (Supplementary Fig. S2). The profiles did not show a consistent correlation between the onset of diarrhoea and remission. Spearman's rank correlation analysis revealed that faecal moisture content was significantly inversely correlated with propionate (*r* = − 0.503, *p* = 0.047) and succinate (*r* = − 0.503, *p* = 0.047), whereas no significant correlations were detected between moisture content and IgA, lactate, butyrate, or total SCFAs (Fig. 1c). Among the individual SCFAs, propionate, butyrate, and acetate were significantly inter-correlated (propionate vs butyrate: *r* = 0.779, *p* < 0.001; propionate vs acetate: *r* = 0.662, *p* = 0.005; butyrate vs acetate: *r* = 0.538, *p* = 0.031) (Fig. 1c). These results suggest that propionate and succinate levels are associated with faecal moisture status, and that the individual SCFAs change coordinately rather than independently. The only metabolite that significantly differed over time was the SCFA butyric acid (Fig. 1d, e). The propionate content appeared to increase after the administration of *Caldifermentibacillus* (Fig. 1e). In addition, IgA levels did not increase consistently after exposure to this thermophile. These results suggest that SCFAs may influence the onset of diarrhoea. Thus, the administration of compost-derived *C. hisashii* to cattle tends to ameliorate preexisting diarrhoea, although the causes of diarrhoea are unclear, and the level of faecal IgA does not consistently increase after exposure to the thermophile.

### Evaluation of faecal bacteria and metabolites for diarrhoeal remission

**Fig. 2:**
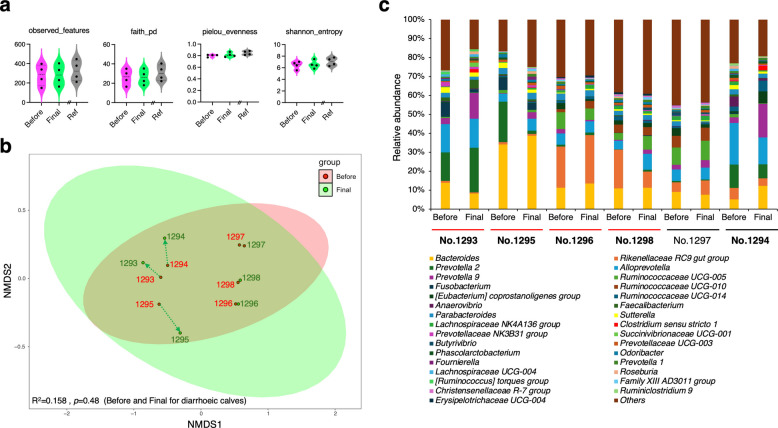
Faecal bacterial diversity in the cattle observed in this study. **a** The observed OTU number, faith values, evenness values, and Shannon indices, as α-diversity indicators, were calculated on the basis of the bacterial data for cattle faeces calculated via QIIME2. “Before” and “Final” indicate the data for No. 1293, No. 1295, No. 1296, and No. 1298, respectively, and “Ref” indicates the data for Nos. 1294 and 1297, without the occurrence of diarrhoea, as described in Supplementary Fig. S1a. These data are described in Supplementary Fig. S1a. **b** Characteristics of the faecal bacteria of the cattle classified via nonmetric multidimensional scaling (NMDS) (stress = 0.043). The NMDS data of the cattle were visualised for evaluation of β diversity. The red and green colours of the individual numbers indicate “Before” and “Final”, respectively, in Supplementary Fig. S1a. The statistical values calculated using the R library ‘pairwiseAdonis’ are shown at the bottom right. **c** The relative abundances of faecal bacterial genera in cattle (1% or more as the maximum relative abundance). The black and red lines indicate without and with the diarrhoeal occurrence, respectively. The bold letters for individual Nos. indicate the administration of *C. hisashii* after “Before” (day 1)

The bacterial diversity before and after the administration of the compost-derived thermophile was evaluated. Four indicators of α diversity were compared between diarrhoeal and non-diarrhoeal states using paired *t*-tests. No significant differences were detected for any index (observed features, *p* = 0.972; faith_pd, *p* = 0.990; pielou_evenness, *p* = 0.569; Shannon entropy, *p* = 0.734) (Fig. 2a). Similarly, β diversity was evaluated by NMDS and tested by PERMANOVA (pairwise.adonis, 999 permutations). The NMDS ordination yielded a stress value of 0.043, indicating a reliable representation of the community structure. No significant separation was detected in any of the comparisons: before-administration (*n* = 7, including the untreated reference After_1297) vs post-administration (*n *= 5) (*R*^2^ = 0.133, *p* = 0.214); diarrhoea vs non-diarrhoea (*R*^2^ = 0.056, *p* = 0.697); or diarrhoeic calves before administration vs recovered calves after administration, excluding untreated references (*n* = 4 each; *R*^2^ = 0.158, *p* = 0.48; Fig. 2b). In addition, bacterial abundance was evaluated at the phylum and genus levels (Supplementary Figs. S3a and Fig. 2c). However, no significant differences were detected at the phylum level (Supplementary Fig. S3a). Also, at the faecal metabolite level, no significant differences in the relative abundances of carbohydrates, phosphate-related metabolites, amino acids, or other acids were detected (Supplementary Fig. S3b).

**Fig. 3:**
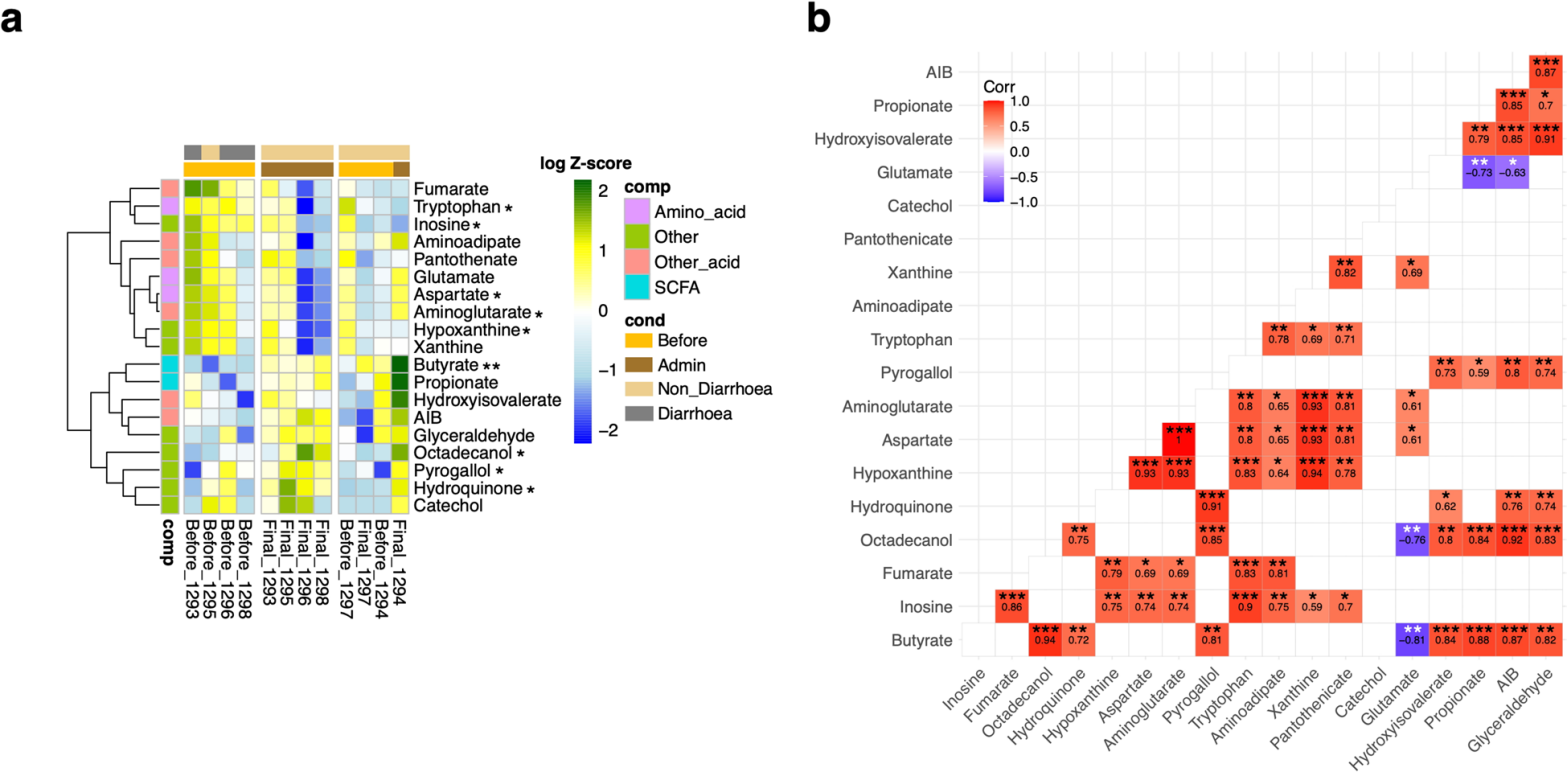
Behavioural evaluation of faecal metabolites in calves with diarrhea. **a** Heatmap of characteristic metabolite groups following diarrhoea onset and remission. Individual profiles of calves were evaluated using log *z*-score-transformed values of characteristic metabolites identified by statistical analysis (*p* < 0.1). **b** Correlation heatmap of these metabolite groups. The abbreviations are as follows: comp, metabolite category; cond, animal management status; Before, prior to probiotic administration; Admin, following administration of *C. hisashii*; Non_Diarrhoea, non-diarrhoeal faecal samples (note: calf No. 1295 showed mild loose faeces on the first day but developed diarrhoea from the following day, with remission observed on day 4 of the administration); Diarrhoea, diarrhoeal faecal samples; AIB, 2_aminoisobutyric acid (aminoisobutyrate); Aminoadipate, 2_aminoadipic acid; Aminoglutarate, 3_aminoglutaric acid; Hydroxyisovalerate, 3_hydroxyisovaleric acid; ***, *p* < 0.001; **, *p* < 0.01; and *, *p* < 0.05

**Fig. 4:**
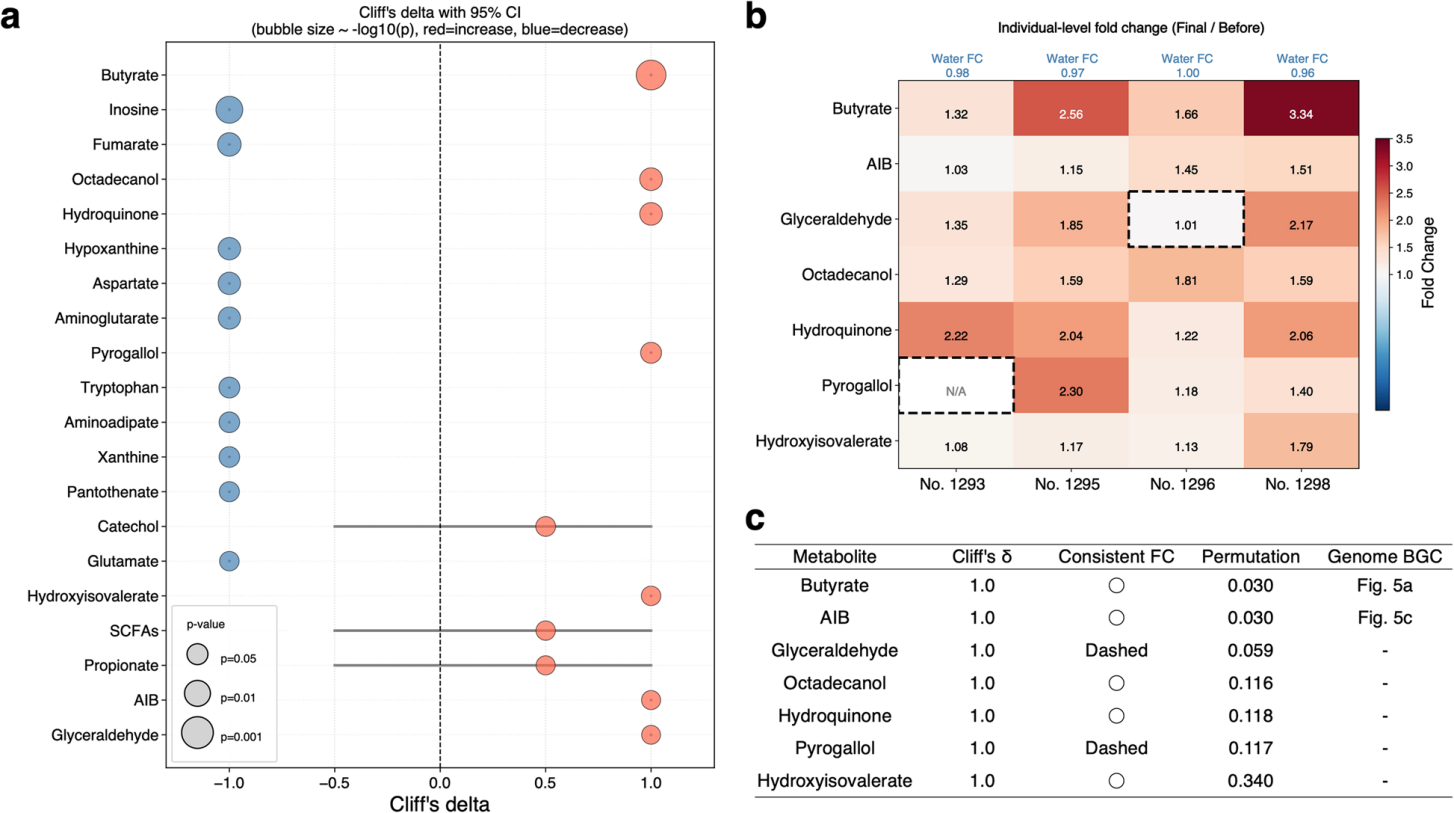
Statistical selection of feature metabolites. **a** Selection of important factors using Cliff's delta. Statistically important metabolite groups (*p* < 0.1) identified in Fig. 3 were further evaluated using Cliff’s delta, a non-parametric effect size measure suitable for small sample sizes. The size of each bubble represents − log10(p). Red and blue indicate increasing and decreasing tendencies, respectively. **b** Individual-level fold changes (Final/Before) for metabolites with Cliff’s delta = 1.0. Dashed boxes indicate inconsistent or incalculable fold changes. Water FC (faecal water content fold change) is shown as the clinical reference for diarrhoeal remission. **c** Evaluation of candidate metabolites with Cliff's delta = 1.0. Column definitions (left to right): Cliff’s δ, Cliff’s delta effect size; consistent FC, consistency of fold change direction across all four diarrhoeic individuals (○, consistent increase; dashed, inconsistent or incalculable, see panel **b**); Permutation, exact *p*-values calculated by enumerating all 70 possible group label permutations using AUC as the test statistic; Genome BGC, figure reference for the corresponding biosynthetic gene cluster identified in *C. hisashii* (–, not identified). Only butyrate and AIB achieved significant exact p-values (*p* < 0.05) with confirmed genome BGC. The abbreviations are as follows: AIB, 2_aminoisobutyric acid (aminoisobutyrate); Aminoadipate, 2_aminoadipic acid; Aminoglutarate, 3_aminoglutaric acid; Hydroxyisovalerate, 3_hydroxyisovaleric acid

Therefore, feature selection to distinguish between diarrhoeal and non-diarrhoeal states was performed using a multi-step statistical approach, including difference-in-differences (DID), Cliff’s delta, and permutation-based validation. As shown in Supplementary Fig. S4, although genera showing increasing or decreasing tendencies (*p* < 0.2) were observed, no statistically significant differences were found in faecal-derived bacterial groups. Seventeen feature bacterial candidates that distinguished between diarrhoeal and non-diarrhoeal states were selected via DID. Among the 19 characteristic metabolites identified (*p* < 0.1) (Fig. 3a and Supplementary Fig. S5), statistically significant differences (*p* < 0.05) were observed in nine components: butyrate (*p* = 0.002195), octadecanol (*p* = 0.028397), aspartate (*p* = 0.031845), aminoglutarate (*p* = 0.033406), hypoxanthine (*p* = 0.03144), inosine (*p* = 0.006768), tryptophan (*p* = 0.049109), hydroquinone (*p* = 0.028528), and pyrogallol (*p* = 0.048318). These metabolites showed relatively strong positive correlations with each other (Fig. 3b). The importance of these metabolites was quantitatively assessed using Cliff’s delta (Fig. 4a), based on two comparisons: diarrhoeal versus non-diarrhoeal status, and probiotic-administered versus non-administered conditions. Among the metabolites with the maximum effect size (Cliff's delta = 1.0), individual-level fold changes were evaluated to confirm the consistency of directional changes across all four diarrhoeic calves (Fig. 4b). To assess whether the observed group separation could arise by chance, exact permutation tests, which are valid regardless of sample size, were performed by enumerating all 70 possible group label assignments (Supplementary Fig. S6). Only butyrate and AIB achieved significant exact *p*-values (exact *p* = 0.029), indicating that their complete separation is unlikely to be a chance finding. Furthermore, among all metabolites with Cliff’s delta = 1.0, butyrate and AIB were the only metabolites for which biosynthetic gene clusters were identified in the *C. hisashii* genome (Fig. 4c). Other candidates with Cliff’s delta = 1.0 (glyceraldehyde, octadecanol, hydroquinone, pyrogallol, and hydroxyisovalerate) are produced via ubiquitous primary metabolic pathways or derived from dietary sources and could not be specifically attributed to *C. hisashii.*

Thus, butyrate and AIB were identified as the primary feature metabolites based on the convergence of statistical, clinical, and mechanistic evidence.

### Evaluation of faecal bacterial metabolic functions associated with diarrhoeal remission

Since no statistically significant differences were observed in faecal bacterial composition at the genus level, PICRUSt2-based functional prediction was performed using faecal 16S rRNA data to explore metabolic functional changes that may reflect cooperative community-level responses associated with diarrhoeal remission. Predicted abundances were subjected to centred log-ratio (CLR) transformation [30] following the recommended procedure for compositional microbiome data: zeros were replaced using Bayesian-multiplicative imputation (zCompositions package in R) prior to CLR transformation (compositions package in R).

At the enzymatic function level (Supplementary Fig. S7 and Supplementary Table S1), 12 EC numbers showed notable changes (*p* < 0.05, Mann–Whitney *U* test). Among these, EC:2.1.1.222 (2-polyprenyl-6-hydroxyphenol methylase), EC:2.1.1.64 (3-demethylubiquinol 3-O-methyltransferase), and EC:2.3.3.14 (homocitrate synthase) showed increasing tendencies following probiotic administration, whereas EC:1.1.5.4 (quinone-dependent malate dehydrogenase) showed a decreasing tendency. The direction of these changes in the probiotic administration comparison mirrored those observed in the diarrhoeal status comparison, suggesting a coherent metabolic shift associated with diarrhoeal remission (Supplementary Table S1).

At the pathway level (Supplementary Table S1), PWY-7187 (pyrimidine deoxyribonucleotides de novo biosynthesis II) was the only pathway reaching significance (*p* < 0.05).

Thus, these findings suggest that metabolic functional shifts at the community level, transcending individual bacterial taxa, may play a role in the process of diarrhoeal remission following *C. hisashii* administration. However, given the small sample size (*n* = 4 per group), these PICRUSt2-based results are presented as exploratory patterns complementing the primary metabolomic and genomic findings.

### Genomic and proteomic evidence for butyrate and AIB production in *Caldifermentibacillus hisashii* N11

To validate the biosynthetic capacity underlying the identified feature metabolites, the complete genome sequence of *C. hisashii* N11 (AP028807.1) was analysed using genome sequence analysis, antiSMASH, and proteomic approaches.

**Fig. 5:**
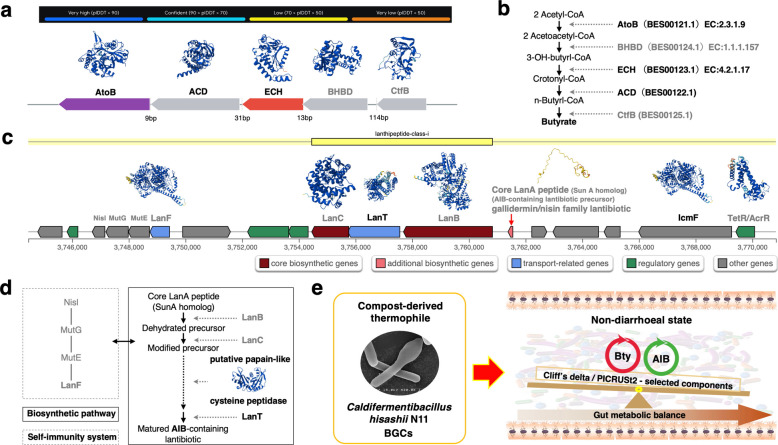
Genomic and proteomic evidence for butyrate and 2-aminoisobutyrate production, and a putative model of probiotic effects in *Caldifermentibacillus hisashii.* **a** Butyrate biosynthetic gene cluster. Genomic organisation of the butyrate biosynthetic gene cluster (BGC) (2,843,472–2,848,237 bp, AP028807.1). Predicted protein structures generated by AlphaFold3 and coloured by pLDDT confidence score (blue, > 90; cyan, 70–90; yellow, 50–70; orange, ≤ 50) are shown above the corresponding genes. Black and grey labels indicate proteins confirmed by supernatant proteomic analysis and those identified at the genome level only (not detected in the proteome), respectively. Abbreviations are defined in Table S2. **b** Proposed cascade for butyrate biosynthesis. Schematic of the butyrate biosynthetic pathway encoded by the gene cluster shown in **a**. **c** Lanthipeptide biosynthetic gene cluster (Region 2). Genomic organisation of the lanthipeptide BGC (3,744,454–3,770,832 bp, AP028807.1) predicted by antiSMASH. Predicted protein structures generated by AlphaFold3 are shown above the corresponding genes and coloured by pLDDT confidence score (as described in **a**). Black and grey labels follow the same convention as in **a**. Abbreviations are defined in Table S3. **d** Proposed cascade for AIB-containing lantibiotic biosynthesis in *C. hisashii*. The biosynthetic pathway shows the sequential modification steps, while the self-immunity system protects the producer cell from mature lantibiotic toxicity. The predicted structure of the DUF1796 family papain-like cysteine peptidase (located 1.74 Mb upstream, confirmed by proteomic analysis) was generated by AlphaFold3 and coloured by pLDDT confidence score (as described in **a**). **e** Proposed model of probiotic effects on the gut microbiota. Conceptual illustration showing that probiotic administration promotes butyrate (Bty) and 2-aminoisobutyrate (AIB) production. The balance of gut microbial metabolic functions, as assessed by PICRUSt2 analyses, is illustrated as key factors modulated by probiotic administration. The abbreviations used in **a**–**d** are as follows: AtoB, acetyl-CoA C-acetyltransferase; ACD, acyl-CoA dehydrogenase family protein; ECH, enoyl-CoA hydratase; BHBD, 3-hydroxybutyryl-CoA dehydrogenase; BGCs, biosynthetic gene clusters; CtfB, CoA transferase subunit B (CotB); LanA, core peptide precursor; LanB, lanthipeptide dehydratase; LanC, lanthipeptide cyclase; LanF, Lantibiotic protection ABC transporter ATP-binding protein; LanT, lantibiotic ABC transporter; IcmF, isobutyryl-CoA mutase fused (annotated as methylmalonyl-CoA mutase family protein in the registered genome); Nisl, NisI/SpaI family lantibiotic immunity lipoprotein; MutG, Lantibiotic immunity ABC transporter permease (MutG family); MutE, Lantibiotic immunity ABC transporter permease (MutE/EpiE family); and TetR/AcrR, TetR/AcrR family transcriptional regulator. See Supplementary Tables S2–S4 for detailed genomic and proteomic information

Regarding butyrate biosynthesis, a dedicated gene cluster (2,843,472–2,848,237 bp) encoding five enzymes involved in the classical butyrate fermentation pathway [31] was identified (Fig. 5a and Supplementary Table S2). Although antiSMASH analysis did not identify the butyrate biosynthetic gene cluster, manual inspection of the genome sequence combined with tblastn searches successfully identified the functionally related genes. The predicted biosynthetic cascade proceeds from acetyl-CoA to butyrate via acetoacetyl-CoA, crotonyl-CoA, and 3-hydroxybutyryl-CoA (Fig. 5b) based on the previously reported pathway [31]. Among the five enzymes, AtoB and ECH were confirmed by supernatant proteomic analysis, while BHBD and CtfB were not detected in the proteome (Supplementary Table S2).

Regarding AIB-containing lantibiotic biosynthesis, antiSMASH predicted a lantibiotic biosynthetic gene cluster (BGC; Region 2, 3,744,454–3,770,832 bp) in the genome of *C. hisashii* N11 (Fig. 5c and Supplementary Table S3). The BGC encodes core biosynthetic enzymes including LanA, LanB, LanC, and LanT, of which LanT was confirmed by proteomic analysis. Furthermore, a putative papain-like cysteine peptidase (DUF1796 family) located 1.74 Mb upstream of the BGC was also confirmed by proteomic analysis, suggesting its role in leader peptide cleavage during lantibiotic maturation (Fig. 5d) based on the previous reports [32, 33]. IcmF (EC:5.4.99.13), encoded within Region 2 and confirmed by proteomic analysis, catalyses the reversible interconversion of isobutyryl-CoA and n-butyryl-CoA, representing a potential metabolic link between butyrate and AIB biosynthesis.

In summary, three BGCs were predicted by antiSMASH in the genome of *C. hisashii* N11, including a terpene BGC (Region 1), the lanthipeptide BGC (Region 2), and a betalactone BGC (Region 3) (Supplementary Table S4). Collectively, these genomic and proteomic findings support a putative model of probiotic effects on the gut microbiota (Fig. 5e), in which *C. hisashii* administration promotes butyrate and AIB production, thereby modulating the balance of gut microbial metabolic functions as assessed by Cliff’s delta and PICRUSt2 analyses.

## Discussion

This study is the first to assess the possibility of diarrhoeal remission caused by a compost-derived thermophile via environmental organic recycling. Physiological protection against diarrhoea was observed in cattle following oral administration of the compost-derived thermophile as a feed additive, despite differences in gut microbiota composition across individual animals (Fig. 1a, b). Notably, faecal water content, IgA, and SCFA levels showed characteristic changes during the remission process (Fig. 1c–e), suggesting that multiple physiological parameters were coordinately modulated during diarrhoeal remission. Feature component candidates for diarrhoeal remission were selected using DID and Cliff’s delta analysis, followed by permutation testing and genomic validation (Fig. 4). Among all candidates with the maximum effect size (Cliff’s delta = 1.0), only butyrate and AIB could be mechanistically attributed to *C. hisashii* through confirmed biosynthetic gene clusters and proteomic evidence. The results of this trial, which enabled short-term remission, reveal a structural feature susceptible to gut microbial metabolism, although the aetiology of diarrhoea in this study was not fully characterised (Supplementary Fig. S1c). The feature components estimated in this study, especially butyrate and AIB, may be associated with diarrhoeal remission irrespective of the specific aetiology.

Regarding gut bacterial diversity, neither α-diversity indices (observed OTU number, Faith’s phylogenetic diversity, evenness, and Shannon index) nor β-diversity (NMDS) showed statistically significant differences between the diarrhoeal and non-diarrhoeal conditions (Fig. 2a, b). These results suggest that diarrhoeal remission in this study was not accompanied by a marked shift in overall bacterial community structure. However, at the genus level, certain bacterial groups, including *Muribaculum*, showed tendencies to increase following *C. hisashii* administration (Fig. 2c). The genus *Muribaculum*, one of the DID-selected components, belongs to the phylum Bacteroidota (synonym: Bacteroidetes). Although not statistically significant, *Muribaculum* abundance showed a slight increasing tendency following *C. hisashii* administration (*p* < 0.2), which is of interest given the previously reported increase in the phylum Bacteroidota following *C. hisashii* administration to cattle, accompanied by increases in faecal SCFA levels and improved growth performance [29]. Notably, *Muribaculum intestinale* has been reported to induce antigen-specific cytokine responses [34] and to restrict *Salmonella* Typhimurium colonisation by converting succinate to propionate [35]. In addition, the genus *Megasphaera*, a bacterium known to utilise lactate and produce SCFAs [36, 37], also showed an increasing tendency under the conditions of this study (Supplementary Fig. S4). Although the host species differed, it is noteworthy that a similar increase was observed in pigs administered compost containing *C. hisashii* [22].

Generally, SCFAs are produced by commensal bacteria [38, 39] and promote peripheral regulatory T-cell generation [40]. An increase in butyrate levels has been observed at moderate temperatures under in vitro artificial anaerobic conditions associated with compost administration [41]. Improvements in animal health via compost-derived thermophiles may be related to the physiological functions of SCFAs, which have been shown to protect gut function in mouse models [42, 43]. Consistent with the role of SCFAs described in previous reports [40, 42–44], increases in faecal butyrate and propionate levels following probiotic exposure (Fig. 1d) may also be important for maintaining homeostatic and metabolic balance (Fig. 5e) during diarrhoeal remission (Fig. 1e).

In general, the causes of diarrhoea are diverse, and associated pathogens include bacteria such as *E. coli*, *Shigella*, *Salmonella*, *Campylobacter*, *Clostridioides difficile*, and *Aeromonas* [5, 45, 46]; fungi such as *Candida* species [5, 47]; and RNA viruses [5, 48]. Research on various prebiotics and probiotics for diarrhoeal remission is ongoing [6, 7]. Prebiotic administration has been shown to increase SCFA levels and enhance intestinal barrier function [49]. Probiotics have also been suggested to produce organic acids and enhance the body’s defence function [50, 51]. Diarrhoeal remission has been observed following improvement of the intestinal microbiota by FMT [8], although some data remain contradictory [9].

In addition to the identification of butyrate and AIB as key feature metabolites, PICRUSt2-based functional prediction suggested further metabolic changes at the community level. At the enzymatic function level, 12 EC numbers showed significant changes (*p* < 0.05), including EC:2.1.1.222 and EC:2.1.1.64 involved in ubiquinone biosynthesis and EC:2.3.3.14 (homocitrate synthase), while EC:1.1.5.4 (quinone-dependent malate dehydrogenase) showed a decreasing tendency (Supplementary Table S1 and Supplementary Fig. S7). Notably, the direction of these changes was consistent between the probiotic administration and diarrhoeal status comparisons, suggesting coherent metabolic shifts at the community level. Although the small sample size (*n* = 4 per group) limits the statistical power of these analyses, these patterns raise the possibility that diarrhoeal remission following *C. hisashii* administration may involve coordinated metabolic shifts across the gut microbial community, beyond the production of individual metabolites such as butyrate and AIB.

To investigate the potential contribution of *C. hisashii* itself to diarrhoeal remission, the complete genome sequence (AP028807.1) was evaluated. A butyrate biosynthetic gene cluster was identified (Fig. 5a and Supplementary Table S2), and several of the encoded enzymes were confirmed by supernatant proteomic analysis. Furthermore, antiSMASH-based BGC prediction identified a lanthipeptide BGC encoding an AIB-containing lantibiotic, consistent with previous reports [52, 53], and several genes within this cluster were partially confirmed by proteomic analysis. Of particular interest, IcmF was identified within this lanthipeptide BGC and confirmed by proteomic analysis. IcmF is known to catalyse the reversible interconversion of isobutyryl-CoA and n-butyryl-CoA [54], suggesting its involvement in an alternative cascade contributing to butyrate biosynthesis.

In addition to the butyrate biosynthetic gene cluster, proteomic analysis identified two components of the branched-chain keto acid dehydrogenase (BKD) complex [55] involved in valine degradation: bfmBB (lipoamide acyltransferase, EC:2.3.1.168; CHN11BP863_21770) and lpdA_2 (dihydrolipoyl dehydrogenase, EC:1.8.1.4; CHN11BP863_21800). Both enzymes were confirmed by supernatant proteomic analysis and catalyse the conversion of valine to isobutyryl-CoA, providing substrate supply for IcmF. Together with IcmF, these findings suggest a metabolic route from valine degradation to butyrate biosynthesis via isobutyryl-CoA interconversion.

Despite the expected biosynthetic pathway for AIB-containing lantibiotics (Fig. 5c–d, and Supplementary Table S3), the canonical maturation enzymes LanB (dehydratase) and LanC (cyclase) were not detected in our proteomic analysis (Supplementary Table S3). This observation can be attributed to the technical challenges associated with detecting and characterising these enzymes. Comprehensive reviews of lanthipeptide biosynthetic enzymes have extensively documented the long-standing technical difficulties in detecting and reconstituting LanB and LanC enzyme activities [32]. Regarding AIB-containing lantibiotic maturation, a DUF1796 family putative papain-like cysteine peptidase (CHN11BP863_18310; BER99297.1; 2,003,731–2,004,360 bp) located 1.74 Mb upstream of Region 2 was confirmed by proteomic analysis (Supplementary Table S3), suggesting its involvement in leader peptide cleavage during lantibiotic maturation. Additionally, a TetR/AcrR family transcriptional regulator [56] was also detected (Supplementary Table S3), consistent with the regulatory machinery typically found in lanthipeptide biosynthetic gene clusters.

According to the literature, AIB is known to be involved in the synthesis of alamethicin and/or lantibiotics [53], which may possess antibacterial [57] and antiviral activities [58] at very low concentrations (ppb levels). Some lantibiotics and cyclic peptides may effectively disrupt viral infectivity [58–60]. Recently, AIB containing peptides with cell membrane permeability have been artificially synthesised [61]. Papain-like proteases have also been reported to be involved in peptide maturation process [62]. Notably, *C. hisashii* harbours a gene encoding a putative papain-like protease in its genome. This gene was confirmed at the protein level by supernatant proteomic analysis (Supplementary Table S3), and fragments consistent with the cleaved peptide were also detected in the *C. hisashii* culture supernatant. Therefore, compost-derived *C. hisashii* may be involved in the synthesis of lantibiotics from AIB.

Notably, *C. hisashii* also harbours a sequence with 94.6% identity to the lantibiotic SunA in its genome (Supplementary Fig. S8). Sequence alignment of the precursor peptides revealed overall sequence identity of 92.9% (52/56 residues), with differences in the leader peptide at positions 18 (D → T) and 19 (V → T), and in the core peptide at positions 19 (V → I) and 36 (R → K). Core peptide identity was 94.6% (35/37 residues). Importantly, AIB residues in matured lantibiotics are generated via post-translational modification of Thr residues by LanB dehydratase, and the *C. hisashii* core peptide contains multiple Thr residues at positions corresponding to those in SunA, suggesting potential AIB incorporation following post-translational modification. However, the results of artificial structural analysis based on the genome database were inaccurate. The predicted structure of lantibiotic LanA1 (https://www.uniprot.org/uniprotkb/Q65DC4/entry), which shares sequence similarity with the SunA-related sequence identified in *C. hisashii*, was not listed as a high-accuracy structure by AlphaFold. Similarly, the predicted structure of the registered SunA itself (https://www.uniprot.org/uniprotkb/A4IJZ5/entry) was also classified as a low-accuracy structure. The sequence identity between the core peptide confirmed in this study and these reference lantibiotics was < 50% for LanA1 and 94.6% for SunA (35 of 37 core peptide residues), suggesting that the lantibiotic produced by *C. hisashii* may share functional properties with SunA. However, accurate structural prediction of this lantibiotic remains challenging, possibly reflecting its nature as a precursor peptide prior to post-translational modification. Additionally, an antibody against an epitope fragment based on the SunA-related peptide sequence was synthesised; however, SunA-related peptides were not detected under the conditions used in this study.

The co-existence of AIB biosynthetic and degradative capacities in *C. hisashii* is not paradoxical but rather reflects a well-established biological principle observed in lantibiotic-producing bacteria. Lantibiotic producers universally possess self-immunity mechanisms, including dedicated immunity proteins and ABC transporters, to protect themselves from the toxic effects of their own products [63]. The balance between production and immunity is tightly regulated through two-component signal transduction systems [64]. As shown in Fig. 5c and Supplementary Table S3, the lanthipeptide BGC of *C. hisashii* N11 encodes multiple self-immunity components, including NisI/SpaI family lipoprotein and ABC transporters (MutE and MutG). Furthermore, the leader peptide of the precursor peptide plays a critical role in preventing autotoxicity prior to maturation by keeping the core peptide inactive until proteolytic cleavage [65, 66], as illustrated in Supplementary Fig. S8. Lantibiotic production itself has also been shown to impose a metabolic burden on producer cells [67], suggesting that degradative pathways may serve as an additional regulatory mechanism to maintain optimal concentrations in the intestinal environment. However, the precise roles of these regulatory mechanisms in *C. hisashii* N11 remain to be experimentally verified. In addition, the genome of *C. hisashii* N11 also encodes a D-β-aminoisobutyrate aminotransferase (D-BAIBAT; EC 2.6.1.44, locus tag CHN11BP863_03220), confirmed by proteomic analysis (Supplementary Table S3). This enzyme is involved in thymine catabolism via degradation of D-β-aminoisobutyrate (BAIBA), which is structurally distinct from the 2-aminoisobutyrate (AIB; α-aminoisobutyric acid; α-AIB) found in lantibiotics, and its relationship to AIB-containing lantibiotic biosynthesis remains unclear.

Notably, AIB was detected in the intestinal environment at low concentrations in cattle as an environmentally derived metabolite, indicating that this amino acid is naturally present in the gut ecosystem. It is conceivable that a portion of the detected AIB may originate from the degradation of AIB-containing lantibiotics produced by *C. hisashii* or other gut microorganisms, although this hypothesis remains to be experimentally verified. The presence of both AIB biosynthetic and degradative pathways in *C. hisashii* suggests that this organism may function as a regulatory factor controlling AIB levels in the intestinal environment, potentially optimising its beneficial effects during diarrhoeal remission.

Regarding other metabolites identified by Cliff’s delta and permutation-based validation, no dedicated biosynthetic gene clusters were identified in the genome of *C. hisashii* N11 for the majority of these feature metabolites, including glyceraldehyde, hydroxyisovalerate, and octadecanol. These metabolites are therefore likely derived from the collective metabolic activities of the gut microbial community rather than being directly produced by *C. hisashii* itself, consistent with the cooperative metabolic model proposed in this study. Their potential roles in intestinal health improvement have been reported as follows: glyceraldehyde can serve as a precursor for glyceraldehyde-3-phosphate, an intermediate in glycolysis [68]. Hydroxyisovalerate is involved in nitrogen metabolism [69] and has also been implicated in inhibiting the growth of colorectal cancer cells [70]. Octadecanol, which acts as an emulsification stabiliser, was reported to be enriched following administration of the probiotic *Lactobacillus casei* Shirota [71].

Among common bacterial interactions, quorum sensing is well known as a mechanism for the control of bacterial populations. For example, probiotic *Bacillus* spp. have been shown to control staphylococcal populations [72]. In this study, compost-derived *C. hisashii* did not exhibit growth inhibitory effects against a strain belonging to the genus *Staphylococcus* (Supplementary Fig. S9a); however, weak antibacterial activity against *E. coli* was observed after long-term cultivation (143.5 h) (Supplementary Fig. S9b). This conditional antibacterial activity is consistent with the genomic identification of an AIB-containing lanthipeptide BGC, suggesting that lantibiotic production by *C. hisashii* may be condition-dependent and contribute to the modulation of gut microbial composition during diarrhoeal remission.

In addition, the results of antiSMASH analysis may be related to the antimicrobial activity observed in Supplementary Fig. S9, which demonstrated the interaction between *C. hisashii* and gut microbiota with selective effects against specific bacterial strains. The antiSMASH analysis also identified terpene precursor and betalactone BGCs (Region 1 and Region 3) apart from the lanthipeptide BGC (Region 2). The functions were not validated in this study; however, these additional mechanisms may allow *C. hisashii* to contribute to gut health beyond the butyrate and AIB production. β-lactones are known to target key metabolic enzymes in fungi and bacteria, including homoserine transacetylase [73], while terpenoids demonstrate broad-spectrum antifungal activities through cell membrane disruption [74]. Importantly, terpene precursors may undergo biotransformation by gut microbiota, generating metabolites with enhanced bioavailability [75]. Given that fungal overgrowth, particularly* Candida* species, can cause secretory diarrhoea in immunocompromised hosts [76], and that neonatal calves face multiple risk factors including immature immunity and potential antibiotic exposure, these putative antifungal capabilities warrant further investigation. Such mechanisms could provide complementary protection against the diverse pathogenic challenges encountered in the calf gut environment, extending beyond the primary metabolic effects we have characterised. 

Finally, the compost-derived thermophile *C. hisashii*, which induced diarrhoeal remission in this study, was identified as an environmental microorganism derived from recycled fermented materials. Although further detailed verification is required, these results suggest that this species represents a natural tool applicable as a countermeasure against the common medical condition of diarrhoea. Recent research under the Zoonomia project [77] has focused on understanding commonalities among animal species, which is expected to be useful not only for understanding ecosystems but also for the development of novel infectious disease control measures within the “One Health” paradigm. These findings may also provide an important perspective for developing “Nature Positive” strategies that promote a harmonious relationship between human society and the natural environment.

## Conclusion

This study shows that oral administration of the compost-derived thermophile *Caldifermentibacillus hisashii* as a feed additive can alleviate diarrhoea in cattle. To clarify the mechanisms of action, discriminative metabolites associated with diarrhoeal remission were selected using DID, Cliff’s delta, and permutation-based validation. These analyses identified a group of candidate metabolites consistently associated with diarrhoeal improvement across individuals with distinct gut microbiota compositions, with butyrate and AIB emerging as the most important discriminative metabolites. Notably, these findings imply that the mechanism underlying diarrhoeal remission may not be attributable solely to individual metabolites, but may also involve cooperative metabolic interactions among the gut microbiota.

Genomic and proteomic analyses of *C. hisashii* confirmed the presence of a butyrate biosynthetic gene cluster and an AIB-containing lanthipeptide biosynthetic gene cluster, providing partial mechanistic support for the protective role of this thermophile against diarrhoeal dysbiosis through the regulation of gut bacterial metabolism. Further studies, including in vivo verification of metabolite production by *C. hisashii* within the bovine gut, will be necessary to fully elucidate these mechanisms. This study contributes to a broader understanding of novel diarrhoea management strategies utilising environmentally derived microorganisms and highlights the importance of considering cooperative metabolic networks within the microbiota as a key mechanism in dysbiosis mitigation.

## Methods

### Cattle management

Cattle rearing trials were conducted at the dedicated farm of Kyushu University, which is located in Oita (N33.05, E131.24). Six Japanese black calves (Nos. 1293, 1294, 1295, 1296, 1297, and 1298) (KC-Test), which were 88.8 ± 8.7 days of age and 103 ± 5.1 kg in body weight (BW) and were born at the Kuju Agricultural Research Center of Kyushu University (Oita, Japan), were used in this study. All the calves were fed a milk substitute of a quality stipulated in the Japanese Feeding Standard for Beef Cattle 2008 (Agriculture, Forestry, and Fishery Research Council Secretariat, MAFF 2008). All the calves were fed calf starter and hay ad libitum according to the Japanese Feeding Standard for Beef Cattle 2000 (Agriculture, Forestry, and Fishery Research Council Secretariat, MAFF 2000). In brief, warm water containing 5 g of potato starch and *C. hisashii* adjusted to 10^2–3^ CFU g^−1^ was administered every morning as total feed to four calves (No. 1293, 1295, 1296, and 1298; the diarrhoea group) that developed diarrhoea and to one calf (No. 1294; the normal group) that did not develop diarrhoea. *C. hisashii* was administered to the diarrhoea group until the calves recovered from diarrhoea (from at least 4 days to 6 days), and it was given to the normal group for 4 days. One calf (No. 1297) was offered warm water for 4 days without *C. hisashii* administration. This duration was set according to our observational data, which revealed that calves recovered from diarrhoea within 4 or 6 days of *C. hisashii* administration. Faeces were collected from the rectum using long gloves immediately from the treated and untreated calves on the first and last days of the treatment period. All animal experiments were carried out according to the guidelines of the farms listed below and the Institutional Animal Care and Use Committee of Chiba University (approval numbers: DOU27-332, DOU28-159, DOU29-174, DOU30-332, DOU1-336, DOU2-341, DOU3-265), Yokohama City University (approval number: T-A-18-003), and Kyushu University (approval number: A29-085-0).

### Preparation of faecal samples

All the faecal samples obtained for the omics analyses were transported at − 20 °C and subsequently stored at − 60 °C to − 80 °C until the subsequent omics analyses were performed. Sequencing of the bacterial 16S RNA gene and metabolomic analyses were performed via the procedures described below.

### Detection of innate and adaptive immune responses

The strength of the immune response was determined to examine the mechanisms of pathogen defence in these animals. Here, the level of faecal immunoglobulin A (IgA), a noninvasive indicator of mucosal immune activity, was measured using an enzyme-linked immunosorbent assay (ELISA) kit according to the manufacturer’s instructions (Bethyl Laboratories Inc., Montgomery, TX, USA).

### Preparation of compost-derived Bacillaceae as a feed additive

Fermented compost from marine animal resources (hereafter, compost) was prepared via an aerobic repeated fed-batch fermentation system at high temperatures (approximately 75 °C) (fermentation-associated self-heating), as previously described [10] (Miroku Co. Ltd., and Keiyo Gas Energy Solution Co. Ltd., Japan). A compost extract was prepared by diluting the compost 1/100 with potable water (in vol./vol.) and incubated under aerobic conditions at 60 °C for at least 10 h [16, 17, 19, 20, 23, 24]. *Caldifermentibacillus hisashii*, a compost-derived Bacillaceae species in the compost solution, was prepared by Sermas Co., Ltd., Japan. *C. hisashii,* which was initially isolated as the *Bacillus thermoamylovorans* N11 strain [25], registered as *B. hisashii* (type strain N-11^ T^ = NRBC 110226^ T^ = LMG 28201^ T^) [26], and under accession number NITE BP-863 given by the international depositary authority at the National Institute of Technology and Evolution (NITE) in Japan (September 26, 2014) and recently reclassified as *C. hisashii* [27], was cultured as previously described [25, 26].

### Detection of bacterial infection

To estimate the cause of diarrhoea in cattle, the presence of pathogenic genes of *Escherichia coli* and *Salmonella enteritidis* was analysed. The primer sets used for detecting the target genes SAA [78], Vtcom [79], VT1 [79], VT2 [79], STx-1 [78], Sta [78], F17 [78], and LT [78] (saa, stx, stx1, sta, f17, and lt, respectively), encoding Shiga toxin in *E. coli*, were used as previously described [78, 79]. A One-Shot PCR Kit (Takara Co., Ltd., Japan) was used to detect the target gene for *S. enteridis* (*invA* gene).

### Diarrhoea remission assessment

The daily occurrence of diarrhoea was recorded for each calf throughout the observation period. To assess whether the temporal pattern of diarrhoea differed significantly across the observation days, Cochran’s *Q *test was performed on the four diarrhoeic calves (Nos. 1293, 1295, 1296, and 1298) with an exact permutation approach. All possible within-row permutations of the binary diarrhoea matrix (days 1–6) were enumerated for each calf. Each C(n,k) represents the number of ways to assign k diarrhoea-positive days within the 6-day observation period: No. 1293, C(6,3); No. 1295, C(6,2); No. 1296, C(6,5); No. 1298, C(6,3), yielding 20 × 15 × 6 × 20 = 36,000 total permutations. The exact *p*-value was calculated as the proportion of permuted Q statistics equal to or exceeding the observed *Q*, without relying on the asymptotic chi-squared approximation, which may be inaccurate at small sample sizes. Analyses were performed using R software (ggplot2, dplyr, tidyr, and scales packages). The implementation of Cochran’s *Q* test and the analysis code are available in the GitHub repository.

### DNA preparation from faecal samples

Total DNA was obtained from faeces according to previously described methods [80] with some modifications. Approximately 100 mg of lyophilised faeces was disrupted with zirconia beads via a Micro Smash MS-100 (Tomy Seiko, Tokyo, Japan) and suspended in 600 μL of a solution containing 10 mM Tris–HCl (pH 8.0) and 1 mM EDTA (TE). The faecal suspension (475 μL) was transferred into a 1.5-mL microcentrifuge tube and incubated with 15 mg/mL lysozyme for 1 h at 37 °C. Achromopeptidase (FUJIFILM Wako Pure Chemical Corp., Osaka, Japan) was added at a final concentration of 2000 units/mL, and the sample was further incubated for 30 min at 37 °C. Proteinase K and sodium dodecyl sulfate (SDS) were added at final concentrations of 1 mg/mL and 1%, respectively, and the mixture was subsequently incubated at 55 °C for 1 h. These enzymatic treatments were carried out with shaking (1000 rpm). The resulting lysate was treated with phenol/chloroform/isoamyl alcohol, and the resulting DNA was precipitated with ethanol. The DNA pellet was rinsed with 70% ethanol, dried, and dissolved in 300 μL of TE. RNase was added at a final concentration of 0.1 mg/mL, and the mixture was incubated at 37 °C for 30 min. An equal volume of a 20% polyethylene glycol 6000 (PEG 6000) solution containing 2.5 M NaCl was subsequently added. After incubation for 30 min on ice, the DNA was pelleted via centrifugation, rinsed, and then dissolved in TE.

### 16S rRNA sequencing and analysis

16S rRNA gene analysis was carried out via PCR amplification with the barcoded forward primer 27F-mod (5′-AGRGTTTGATYMTGGCTCAG-3′) and reverse primer 338R (5′TGCTGCCTCCCGTAGGAGT-3′) primers targeting the hypervariable regions (V1–V2) of the 16S rRNA gene according to a previous report [80]. The PCR mixture comprised 4 µL of DNA template, 0.5 µL of ExTaq polymerase (Takara Bio Inc., Kusatsu, Japan), 5 µL of ExTaq buffer, 5 µL of dNTPs, and 10 pmol each of the forward and reverse primers and was brought to a total volume of 50 µL with DNA-free water. Amplicons were generated with 30 cycles of 98 °C for 30 s, 55 °C for 45 s, and 72 °C for 2 min and were subsequently purified using AMPure XP (Beckman Coulter Inc., Brea, CA, USA). Multiplexed amplicon sequences for the samples were sequenced via the MiSeq platform according to the manufacturer’s instructions. The obtained FASTAQ format files were analysed using QIIME2 (https://qiime2.org) [81]. In addition, the sequences were trimmed via maxEE (default: inf) of the DADA2 plugin, and the trimmed sequences were taxonomically classified via vsearch (using the SILVA database). All 16S rRNA sequence datasets were deposited in the GenBank Sequence Read Archive database, as described in the “Data and materials availability” section. The statistical analysis of nonmetric multidimensional scaling (NMDS) values was performed using the “vegan” (distance = bray as the calculation condition) and “pairwiseAdonis” libraries in R software. The plot was visualised using the “ggplot2” library. Functional prediction of the gut microbial community was performed using PICRUSt2 (Phylogenetic Investigation of Communities by Reconstruction of Unobserved States 2) [82] based on the 16S rRNA amplicon data. The predicted functional abundances were subjected to centred log-ratio (CLR) transformation using Bayesian-multiplicative zero replacement (zCompositions package) and the compositions package in R [30]. Group comparisons were conducted using the Mann–Whitney *U* test (two-sided), implemented using the scipy library in Python. Log_2_ fold changes were calculated as log_2_ (mean of group 2/mean of group 1). Two primary comparisons were performed: diarrhoeal versus non-diarrhoeal status, and probiotic-administered versus non-administered conditions. Only results with *p* < 0.05 are reported. Individual-level changes in EC numbers with significant differences (*p* < 0.05) were visualised as dot plots using matplotlib, in which each animal was represented by a distinct marker shape and group differences were indicated by colour. Mean values ± standard error of the mean are shown. Given the small sample size (*n *= 4 per group), these results are presented as exploratory patterns.

### Metabolite analysis

The concentrations of SCFAs (acetate, propionate, butyrate), lactate, and succinate in the frozen fresh faecal samples were determined using an HPLC Prominence instrument equipped with an electrical conductivity detector (CDD-10A_VP_) (Shimazu, Japan) [41] according to the manufacturer’s protocol with some modifications. In brief, faecal samples (200–400 mg) were mixed with 9 volumes of Milli-Q water (by volume) for 10 min. After centrifugation at 15,000 rpm, the supernatant was filtered with a 0.45 μm Millex-HA filter (SLHA033SS) (Millipore, USA). The filtered solutions were prepared for HPLC analysis. HPLC analysis was carried out on an ion-exclusion column (Shim-pack SCR-102H) (Shimazu, Japan). The measurement conditions were as follows: mobile phase, 5 mM *p*-toluenesulfonic acid; buffer, 5 mM *p*-toluenesulfonic acid, 20 mM Bis–Tris, and 0.2 mM EDTA-4H; temperature setting of the instrument, 40 °C; and flow rate, 0.8 mL/min.

The other water-soluble metabolites in the faeces and culture samples were determined via gas chromatography‒tandem mass spectrometry (GC‒MS/MS) platforms according to the methods described by Nishiumi et al*.* [83] with some modifications. Lyophilised faeces obtained from the caecum were disrupted with zirconia beads via a Micro Smash MS-100 (Tomy Seiko, Tokyo, Japan). The resulting freeze-dried 5 mg faecal samples were suspended in 150 μL of Milli-Q water containing an internal standard (100 μmol/L 2-isopropylmalic acid), and then 150 μL of methanol and 60 μL of chloroform were added. These samples were homogenised and incubated at 37 °C for 30 min with shaking at 1200 rpm. After centrifugation at 16,000 × *g* for 5 min at room temperature, 250 μL of the supernatant was transferred to a new tube, and 200 μL of Milli-Q water was added. After mixing, the mixture was centrifuged at 16,000 × *g* for 5 min at room temperature, and 250 μL of the supernatant was transferred to a new tube. The samples were evaporated to dryness via a vacuum evaporator system (CentriVap Centrifugal Vacuum Concentrator) (LABCONCO, USA) for 20 min at 40 °C and lyophilised via a freeze dryer (TAITEC, Japan). The dried extracts were first methoxylated with 40 μL of 20 mg/mL methoxyamine hydrochloride (Sigma‒Aldrich) dissolved in pyridine. After the derivatisation agent was added, the samples were shaken at 1200 rpm for 90 min at 30 °C. The samples were then silylated with 20 μL of N-methyl-N-trimethylsilyl-trifluoroacetamide (MSTFA) (GL Science, Japan) for 30 min at 37 °C with shaking at 1200 rpm. After derivatisation, the samples were centrifuged at 16,000 × *g* for 5 min at room temperature, and the supernatant was transferred to a glass vial for GC/MS/MS measurements. GC‒MS/MS analysis was carried out on a Shimadzu GCMS-TQ8030 triple quadrupole mass spectrometer (Shimadzu, Japan) with a capillary column (BPX5) (SGE Analytical Science, Australia). The GC oven was programmed as follows: 60 °C for 2 min, increased to 330 °C at 15 °C/min, and finally held at 330 °C for 3.45 min. GC was performed in constant linear velocity mode set at 39 cm/sec. The detector and injector temperatures were 200 and 250 °C, respectively. The injection volume was set to 1 μL with a split ratio of 1:30. Data processing was performed using LabSolutions Insight software (Shimadzu).

### Feature selection

To identify feature metabolites associated with diarrhoeal remission, a multi-step statistical approach was applied. First, paired *t*-tests were performed on raw metabolite data to screen candidate variables (*p* < 0.10). Subsequently, Cliff’s delta [84], a non-parametric effect size measure suitable for small sample sizes, was calculated using log-transformed data to evaluate the magnitude and direction of changes between before and after probiotic administration. Cliff’s delta values were estimated with 95% confidence intervals via bootstrap resampling (*n* = 10,000 iterations), and variables with |δ|≥ 0.474 (large effect size) [85] whose confidence intervals did not include zero were selected as feature candidates. To assess whether the observed group separation exceeded that expected under the null hypothesis, exact permutation tests were performed by enumerating all possible group label assignments. C(n,k) represents the number of ways to assign k samples to the Before group from n total samples: C(8,4) = 70 combinations (4 before and 4 final samples from 8 total). The area under the receiver operating characteristic curve (AUC) was used solely as a test statistic within the permutation framework, not as a selection criterion. The exact p-value was calculated as the proportion of permutations yielding AUC values equal to or exceeding the observed AUC, without relying on asymptotic approximations. Metabolites with permutation *p* < 0.05 and confirmed biosynthetic gene clusters in *C. hisashii* were considered as primary feature metabolite candidates.

### Genomic analysis

The genomic DNA of *C. hisashii* was prepared according to a previously reported method [86]. Briefly, bacterial cells were incubated with 15 mg/ml lysozyme and 2000 units/ml achromopeptidase and then treated with 1% SDS and 1 mg/ml proteinase K. Genomic DNA was extracted with phenol–chloroform–isoamyl alcohol, isolated by ethanol precipitation, and purified by RNase treatment, followed by precipitation with polyethylene glycol 6000 (PEG 6000). Whole-genome sequencing was performed using the PacBio Sequel II system (Pacific Biosciences, California, Inc., USA [PacBio]). The library was prepared with the SMRTbell Express Template Preparation Kit 2.0 (PacBio) with DNA shearing performed using a gTUBE (Covaris, LLC, USA) and a target length of 10–15 kb. The PacBio reads were converted to circular consensus sequencing (HiFi) reads using the ccs software (https://github.com/PacificBiosciences/ccs; v.6.2.0) and assembled using the Canu assembler (v.2.1.1) with specified parameters (minReadLength = 2200; minOverlapLength = 2200) [87]. The HiFi reads were remapped to the generated contigs via Minimap2 (v2.24-r1122) [88], and those contigs with < 5 × coverage were removed. The generated circular contigs were checked for circularisation to remap the HiFi reads, and the linear contigs that mapped to the circular chromosome with ≥ 99% identity and ≥ 95% coverage were removed as bubbles. The obtained genomic sequence was annotated via the DDBJ Fast Annotation and Submission Tool (DFAST v1.2.18, https://dfast.nig.ac.jp) [89]. The complete genome sequence of *Caldifermentibacillus hisashii* N11 [26] was registered as AP028807.1 and used for the following analyses.

### Biosynthetic gene cluster prediction

Biosynthetic gene clusters (BGCs) were predicted using antiSMASH (v8.0.4; https://antismash.secondarymetabolites.org) [90, 91]. The butyrate biosynthetic gene cluster was identified by manual inspection of the genome sequence using the annotated FASTA format genome sequence (.fasta) and predicted protein sequences (.faa) derived from the registered genome (AP028807.1). Candidate genes encoding butyrate biosynthetic enzymes were identified by sequence similarity searches using tblastn against the registered genome sequence, and the results were cross-referenced with the genome annotation in the feature table. The complete genome sequence of *Caldifermentibacillus hisashii* N11 (AP028807.1) was used for all genomic analyses. Biosynthetic gene clusters (BGCs) were predicted using antiSMASH. The butyrate biosynthetic gene cluster was identified by screening the annotated feature table of AP028807.1 for genes encoding butyrate biosynthetic enzymes, followed by retrieval of the corresponding protein sequences using NCBI Entrez E-utilities (efetch). Candidate genes were confirmed by tblastn searches (NCBI BLAST + suite) against the genome sequence and cross-referenced with the NCBI feature table annotation. Identified genes were further validated against available proteomic data to confirm protein-level expression. Genomic organisation of the identified cluster was visualised using custom Python scripts (matplotlib). Full details of the identified genes and protein sequences are provided in Supplementary Tables S1 and S2.

### Chemical and protein structure prediction

Target gene sequences in the complete genome of *Caldifermentibacillus hisashii* N11 (AP028807.1) [26] were translated into amino acid sequences, and protein structures were predicted using AlphaFold2 (https://alphafold.ebi.ac.uk) [92, 93] for initial screening and AlphaFold3 (https://alphafoldserver.com) [94] for final structure prediction.

### Proteomics

To verify the actual presence of these proteins, a proteomic analysis of the culture supernatant of *C. hisashii* was performed using a previously reported method [95, 96] with some modification. In brief, approximately 100 ml of the supernatant was applied to the Amicon Ultra ultrafiltration unit (molecular cut-off 3 kDa, Merck Millipore). Proteins on the membrane filter were then reduced, alkylated and digested with trypsin as previously described [97]. Peptides were collected by centrifugation and purified using SCX (GL Science, Co., Ltd.) and Stage tip #84,850 (Thermo Fisher Scientific, USA). The eluted peptides were then dried by vacuum centrifugation and dissolved in 5% ACN containing 0.1% trifluoroacetic acid. The trypsin-digested peptides were analysed on an Orbitrap QE plus (Thermo Fisher Scientific, USA) using nano liquid chromatography (EASY-nLC 1200; Thermo Fisher Scientific; Yasir et al*.*, 2022). The purified peptides were loaded and separated on an Aurora column (25 cm × 75 µm ID, 1.6 mm C18; Ionoptics) with a linear acetonitrile gradient (0–35%) in 0.1% formic acid at a flow rate of 300 nL min-1. Peptide ions were detected by Orbitrap QE plus MS (Thermo Fisher Scientific, USA) in data-dependent acquisition mode using the installed Xcalibur software (Thermo Fisher Scientific, USA). Full scan mass spectra were acquired in the MS over 375–1500 m/z with a resolution of 70,000. MS/MS searches were performed using PEAKS Studio 10.6 (Bioinformatics Solutions Inc., USA). Mass tolerances in MS and MS/MS were set to 10 ppm and 0.6 Da, respectively. Trypsin was specified as the protease and a maximum of two missed cleavages were allowed. Peptide identification: − 10 lgP > 30.

### Establishment of the antibody

An antibody against the peptide (an antimicrobial lantibiotic precursor peptide) based on the sequence for SunA, an antibiotic-related gene, was established. On the basis of the SunA-like sequence and the sequence of the gallidermin/nisin family lantibiotics (accession no. WP_082026128.1), the sequence of the targeted epitope was CDFDLDIVVKKQDTTV-amide. The C-terminus was amidated to suppress the production of end-recognised antibodies. The sequence itself contains a moderately hydrophilic amino group, and a partial turn structure is predicted as a secondary structure. The overall sequence was determined to include a sequence with high antigenicity. Antibodies were produced in one SPF-grade rabbit four times over a period of 56 days as follows: day 0, preliminary blood sampling and antigen administration; day 14, antigen administration; day 28, antigen administration; day 42, antigen administration; and day 56, whole blood collection. Production was conducted by Sigma-Aldrich Japan Ltd., Japan. Immunoblots were performed to detect SunA. Briefly, the extracts from the compost were separated by electrophoresis on 10% SDS polyacrylamide gels and then transferred to PVDF membranes (GE Healthcare Life Sciences, Sweden). The membranes were blocked with PVDF Blocking Reagent (TOYOBO, Japan) at 4 °C overnight. The primary antibodies used were a rabbit polyclonal anti-MN908947 post-3 antibody diluted 1:3000 or a rabbit polyclonal anti-SunA antibody diluted 1:3000, and the secondary antibody was a horseradish peroxidase-conjugated goat anti-rabbit IgG antibody diluted 1:6000 (Cell Signaling Technology, USA). The immunoblots were incubated with primary and peroxidase-conjugated secondary antibodies at room temperature for 1 h. The protein signals were detected using Western Lightning™ ECL Pro (Perkin-Elmer Life Sciences, USA).

### In vitro halo assay

In a 5-mL culture tube, 50 µl of the culture medium of each strain was incubated in GAM liquid media under anaerobic conditions at 37 °C for 18.5 h. *Enterococcus gallinarum* JCM8728, *Streptococcus equinus* JCM7879, *Streptococcus alactolyticus* JCM31116, *Bifidobacterium longum*, and *Escherichia coli* K12 were used as the test strains. Each strain was spread on GAM agar media. The culture supernatants, vegetative cells, and spores of *Caldifermentibacillus hisashii* N11 were absorbed onto sterile filter paper. The filters were placed on bacterium-infused GAM agar plates under anaerobic conditions at 37 °C. The plates were incubated for 143.5 h, except for the plate containing *Bifidobacterium*, which was incubated for 120 h. The degree of halo formation was also evaluated. Ampicillin (100 mg/mL) was used for a positive control experiment.

### Statistical analyses

Data were analysed as follows. Difference-in-differences (DID) analysis was used to account for individual baseline variation and identify variables showing differential changes between groups. The Shapiro–Wilk test was used to evaluate normality and to select appropriate parametric or non-parametric analyses, performed using the built-in “shapiro.test” function and the “MVN” library in R software. For paired comparisons between two time points using the same individuals, paired *t*-tests were used as the primary statistical test for parametric data, and the Wilcoxon signed-rank test was used for non-parametric data. For time-series data with multiple time points from the same individuals (SCFA concentrations, faecal water content, and IgA levels), repeated measures ANOVA followed by post-hoc paired *t*-tests with Bonferroni correction was applied for parametric data, and the Friedman test followed by post-hoc Wilcoxon signed-rank tests with Bonferroni correction was applied for non-parametric data. Spearman’s rank correlation coefficients were calculated and visualised using the “corrplot” library in R software (method = “spearman”). For metabolite feature selection, normality was assessed using the Shapiro–Wilk test (scipy.stats.shapiro) and equality of variances was evaluated using the Levene test (scipy.stats.levene), implemented in Python. Paired *t*-tests (scipy.stats.ttest_rel) were performed as the primary statistical test to screen candidate variables (*p* < 0.10). The Wilcoxon signed-rank test (scipy.stats.wilcoxon) was additionally performed as a reference non-parametric test; however, owing to the small sample size (*n* < 6), the minimum achievable *p*-value was 0.125. Statistical significance was declared at *p* < 0.05, and a trend was considered at 0.05 ≤ *p* < 0.10. All data are presented as means ± SEM. Analyses were performed using R software (version 4.3.3), Prism software (version 10.0.1), Microsoft Office (version 16.66.1), and Python (scipy, numpy, scikit-learn, and statsmodels libraries). Data collection and analyses were not performed in a blinded manner. In addition, an AI-assisted tool (Claude, Anthropic) was used to support the development of Python and R scripts for statistical analysis and data visualisation. All the codes were critically reviewed and validated for the accuracy and integrity of the final scripts and results.

## Supplementary Information

Supplementary Material 1. Figure S1. Conditions associated with calf diarrhoeal outbreaks. Figure S2. Time course in the contents of water, IgA, SCFAs, lactate, and succinate in the faeces of calves.Figure S3. B Relative abundance of the faecal bacterial phyla and their major metabolites in calves. Figure S4. Relative abundance of the faecal bacterial genera in calves. Figure S5. Alterations in the faecal feature metabolites in calves. Figure S6. E Permutation test results for candidate feature metabolites with Cliff’s delta = 1.0 in calves. Figure S7. Evaluation of gut bacterial metabolic function via the faecal 16S rRNA-based PICRUSt2 analysis in calves. Figure S8. Sequence alignment of precursor peptides of Caldifermentibacillus hisashii N11 and SunA (A4IJZ5). Figure S9. Antibacterial test of Caldifermentibacillus hisashii. Table S1. Differentially predicted functions (EC numbers and pathways) identified by faecal 16S rRNA-based PICRUSt2 analysis in calves. Table S2. Identification of genome-encoded genes involved in butyrate biosynthesis. Table S3. Identification of genome-encoded genes involved in AIB-containing lantibiotic biosynthesis and self-immunity. Table S4. Identification of biosynthetic gene clusters (BGCs) in Caldifermentibacillus hisashii N11.

Supplementary Material 2.

## Data Availability

Raw files of the bacterial V1–V2 16S rRNA data were deposited in the DNA Data Bank of Japan (DDBJ) under NCBI BioProject accession number PRJDB9535 (BioSample Accession no. SAMD00233394–SAMD00233405). Raw files of the bacterial whole-genome data for the Caldifermentibacillus hisashii N11 strain were deposited in DDBJ under the NCBI BioProject accession number PRJDB16338 (BioSample Accession no. SAMD00635923), and the associated BioSample record SAMD00635923_N11 (chromosome, AP028807; pN11a, AP028808; and pN11b, AP028809). Raw files of metabolite profiling of bacterial culture samples and animal faecal samples were deposited in the China National Center for Bioinformation/Beijing Institute of Genomics, Chinese Academy of Sciences (NGDC) under the NGDC OMIX Bioproject accession number PRJCA044254 (OMIX Accession no. OMIX011314, OMIX011375, OMIX011407, and OMIX011408). Proteomics data from the supernatant culture of Caldifermentibacillus hisashii N11 strain were deposited in the JPOST (Japan ProteOme Standard) repository under the dataset identifier JPST004004 (ProteomeXchange ID: PXD067284) (https://repository.jpostdb.org/entry/JPST004004) and JPST004529 (ProteomeXchange ID: PXD076294) (https://repository.jpostdb.org/entry/JPST004529). The relevant protocol was described in the Supplementary Method. A source data file (“Data_Cattle_final.xlsx”) and the additional supplementary raw data files are provided on the figshare site (https://doi.org/10.6084/m9.figshare.31881799). The source commands are provided on the GitHub site (https://github.com/hmiyamoto2000/Cattle_Analysis), and the DOI is https://doi.org/10.5281/zenodo.20319382. Please contact the corresponding authors to obtain any additional information.

## Abbreviations

SCFA: Short chain fatty acid
ROC: Receiver operating characteristic
AUC: The area under the curve
