# Supplementary material for: Gut-protective metabolic phenotype for diarrhoeal remission caused by an environmental probiotic thermophile
Source: Microbiome. 2026 Aug 5;14:194. doi: 10.1186/s40168-026-02476-9 (PMC13445920; doi:10.1186/s40168-026-02476-9)
Supplement: Supplementary file 2 [file 40168_2026_2476_MOESM2_ESM.pdf]

Supplementary Information

Gut-protective metabolic phenotype for diarrhoeal remission caused by an environmental probiotic thermophile

Hirokuni Miyamoto[1,2,3,4,5]*†, Hideyuki Takahashi[6]*†, Wataru Suda[1], Haruki Yamano[1,6], Yudai Inabu[7], Hiroaki Kodama[1], Yumiko Nakanishi[1], Shigeharu Moriya[8], Takashi Satoh[9], Tamotsu Kato[1], Chie Shindo[1], Naoko Tsuji[4], Makiko Matsuura[2,4], Chitose Ishii[1,4], Teruno Nakaguma[2,3], Tetsuji Etoh[6], Yuji Shiotsuka[6], Motoaki Udagawa[10], Atsushi Kurotani[11], Kenta Suzuki[12], Hiroshi Masuya[12], Satoshi Wada[8], Shinji Fukuda[1,2,13,14,15,16], Yukihiro Tashiro[17,18], Hisashi Miyamoto[4,19], Jun Kikuchi[5,20], Masahira Hattori[1,21], Takumi Nishiuchi[22], Naoki Yamamoto[23], Hiroshi Ohno[1]*

[1]RIKEN Center for Integrative Medical Sciences, Yokohama, Kanagawa 230-0045, Japan
[2]Graduate School of Horticulture, Chiba University, Matsudo, Chiba 271-8501, Japan
[3]Japan Eco-science (Nikkan Kagaku) Co., Ltd., Chiba, Chiba 263-8522, Japan
[4]Sermas Co., Ltd., Ichikawa, Chiba 272-0033, Japan
[5]Graduate School of Medical Life Science, Yokohama City University, Yokohama, Kanagawa 230-0045, Japan
[6]Kuju Agricultural Research Center, Kyushu University, Takeda, Oita 878-0201, Japan
[7]Graduate School of Integrated Sciences for Life, Hiroshima University, Higashihiroshima, Hiroshima 739-8528, Japan
[8]RIKEN Center for Advanced Photonics, Wako, Saitama 351-0198, Japan
[9]Division of Hematology, Kitasato University School of Allied Health Sciences, Sagamihara, Kanagawa 252-0373, Japan
[10]Keiyo Gas Energy Solution Co. Ltd., Ichikawa, Chiba 272-0033, Japan
[11]Research Center for Agricultural Information Technology, National Agriculture and Food Research Organization, Tsukuba, Ibaraki 305-0856, Japan
[12]*RIKEN, BioResource Research Center, Tsukuba, Ibaraki 305-0074, Japan*
[13]Institute for Advanced Biosciences, Keio University, Tsuruoka, Yamagata 997-0052, Japan
[14]Gut Environmental Design Group, Kanagawa Institute of Industrial Science and Technology, Kawasaki, Kanagawa 210-0821, Japan
[15]Transborder Medical Research Center, Institute of Medicine, University of Tsukuba, Tsukuba, Ibaraki 305-8575, Japan
[16]Innovative Microbiome Therapy Research Center, Juntendo University Graduate School of Medicine, Tokyo 113-8421, Japan
[17]Laboratory of Soil and Environmental Microbiology, Division of Systems Bioengineering, Department of Bioscience and Biotechnology, Faculty of Agriculture, Graduate School of Bioresources and Bioenvironmental Sciences, Kyushu University, Fukuoka 819-0395, Japan
[18]Laboratory of Microbial Environmental Protection, Tropical Microbiology Unit, Center for International Education and Research of Agriculture, Faculty of Agriculture, Kyushu University, Fukuoka 819-0395, Japan
[19]Miroku Co. Ltd., Kitsuki, Oita 873-0021, Japan
[20]RIKEN Center for Sustainable Resource Science, Yokohama, Kanagawa 230-0045, Japan
[21]School of Advanced Science and Engineering, Waseda University, Shinju-ku, Tokyo169-8555, Japan
[22]Division of Integrated Omics Research, Bioscience Core Facility, Research Center for Experimental Modeling of Human Disease, Kanazawa University, Kanazawa, Ishikawa 920-8640, Japan
[23]Japan Institute for Health Security, Shinjuku-ku, Tokyo 162-8655, Japan

# Co-first Author * Correspondence

Correspondence: Hirokuni Miyamoto, Ph.D., Chiba University, RIKEN

Correspondence: Hideyuki Takahashi, Ph.D., Kyushu University

Correspondence: Hiroshi Ohno, M.D. & Ph.D., RIKEN IMS

Supplementary Figure Captions

Fig.S1. Conditions associated with calf diarrhoeal outbreaks.

Fig.S2. Time course in the contents of water, IgA, SCFAs, lactate, and succinate in the faeces of calves.

Fig.S3. Relative abundance of the faecal bacterial phyla and their major metabolites in calves.

Fig.S4. Relative abundance of the faecal bacterial genera in calves.

Fig.S5. Alterations in the faecal feature metabolites in calves.

Fig.S6. Permutation test results for candidate feature metabolites with Cliff's delta = 1.0 in calves.

Fig.S7. Evaluation of gut bacterial metabolic function via the faecal 16S rRNA-based PICRUSt2 analysis in calves.

Fig.S8. Sequence alignment of precursor peptides of *Caldifermentibacillus hisashii* N11 and SunA (A4IJZ5).

Fig.S9. Antibacterial test of *Caldifermentibacillus hisashii*.

Supplementary Table Captions

Table S1. Differentially predicted functions (EC numbers and pathways) identified by faecal 16S rRNA-based PICRUSt2 analysis in calves.

Table S2. Identification of genome-encoded genes involved in butyrate biosynthesis.

Table S3. Identification of genome-encoded genes involved in AIB-containing lantibiotic biosynthesis and self-immunity.

Table S4 Identification of biosynthetic gene clusters (BGCs) in *Caldifermentibacillus hisashii* N11

a

Target	Group	Calf no.	Age (day)	Tested days	Diarrhoea	*Caldithermo bacillus*	Category
Ref (healthy)	Non-treated	1297	not checked	Day1	-	non_CB	Before
				Day2	-	non_CB	Next
				Day3	-	non_CB	Semifinal
				Day4	-	non_CB	Final (After)
		1294	77	Day1*	-	non_CB	Before
			78	Day2	-	CB	Next
			79	Day3*	-	CB	Semifinal
			80	Day4*	-	CB	Final (After)
Diarrhoea	*Caldibacillus*	1293	103	Day1*	+	non_CB	Before
			104	Day2	+	CB	Next
			105	Day3*	+	CB	Semifinal
			106	Day4*	-	CB	Final (After)
		1295	74	Day1*	-	non_CB	Before
			75	Day2	+	CB	Next
			76	Day3*	+	CB	Semifinal
			77	Day4*	-	CB	Final (After)
		1296	116	Day1	+	non_CB	Before
			117	Day2	+	CB	Next
			118	Day3	+	CB	Day3
			119	Day4	+	CB	Day4
			120	Day5	+	CB	Semifinal
			121	Day6	-	CB	Final (After)
		1298	74	Day1	+	non_CB	Before
			75	Day2	+	CB	Next
			76	Day3	+	CB	Semifinal
			77	Day4	-	CB	Final (After)

b

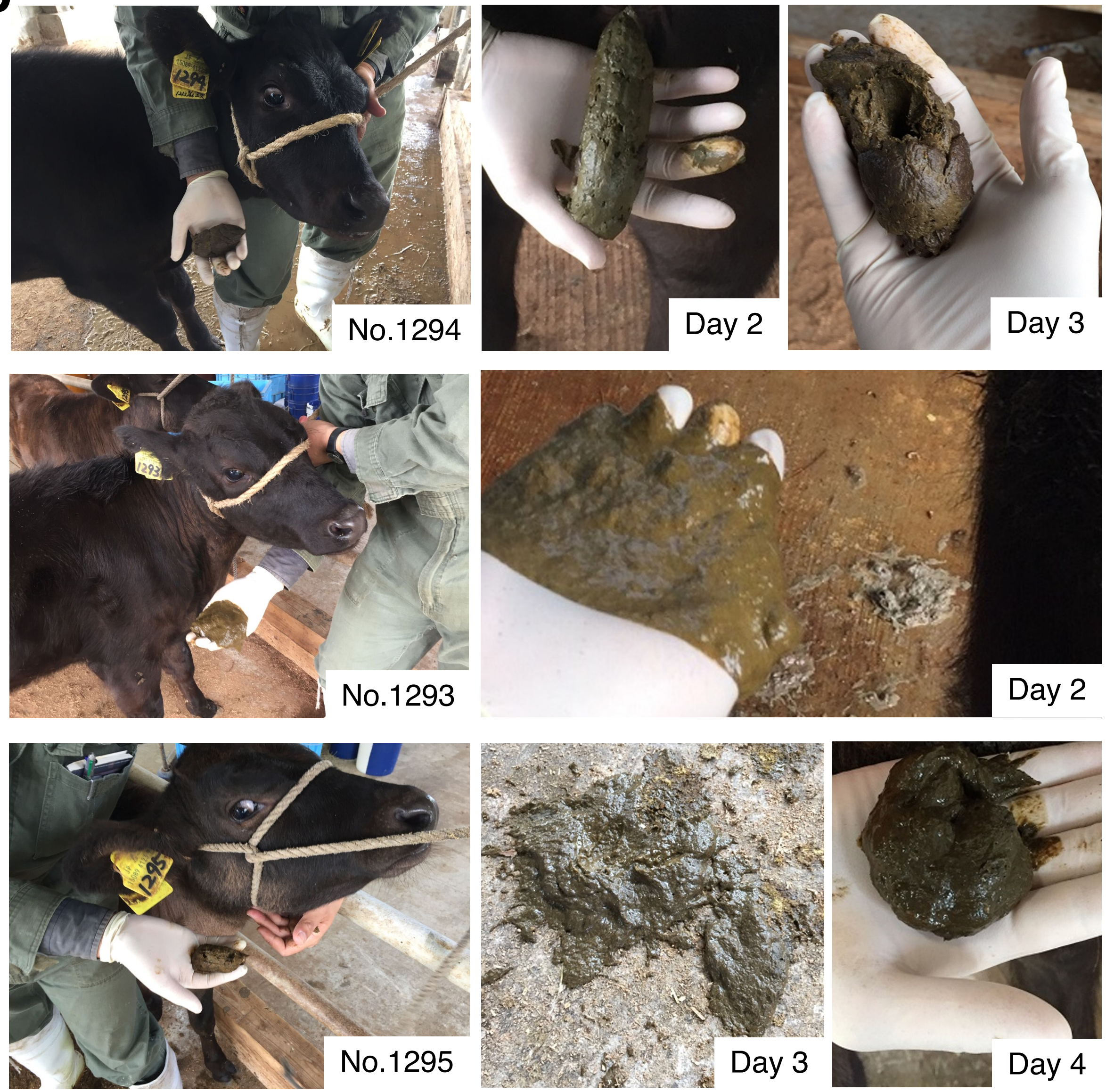

c

Pathogenic genes			Calf no. in Cattle Test					
Origin	Gene name/ primer name		1297	1294*	1293*	1295*	1296	1298
Pathogenic *E. coli*	saa	SAA	x	x	○	x	x	x
		Vt com	x	x	○	○	x	x
	stx	VT1	○	x	○	○	x	x
		VT2	○	x	x	x	x	x
	stx1	STx-1	○	x	○	○	x	○
	sta	STa	○	x	x	x	○	○
	f17	F17	○	○	○	○	○	○
	lt	LT	○	○	○	○	○	○
Salmonella sp.	inv A		x	x	x	x	x	x

Fig.S1 Conditions associated with calf diarrhoeal outbreaks

(**a**) Presence or absence of diarrhoea by individual and its clinical course. (**b**) Photographs of selected individual calves, indicated by an asterisk in the table in Fig. S1a. (**c**) Detection of pathogenic genes of *Escherichia coli* and *Salmonella* sp. in faecal samples from calves on Day 0 (n = 6). The gene names and corresponding primer sequences for Shiga toxin-producing *E. coli* and *Salmonella* pathogenic genes are provided in the Supplementary Materials. Calves No. 1297 and No. 1294 did not develop diarrhoea. The symbols "〇" and "×" indicate positive and negative detection results for the targeted pathogenic genes, respectively. These data were analysed using the chi-square test ($\chi^2 = 0$, df = 1, $p = 1$). The abbreviations are as follows: not treated, normal breeding management without any intervention; not checked, age on the day of the experiment could not be accurately determined (approximately 2 months of age); *, individual photographed; +, diarrhoea occurred; –, no diarrhoea observed; non, probiotic not administered; ad, probiotic orally administered.

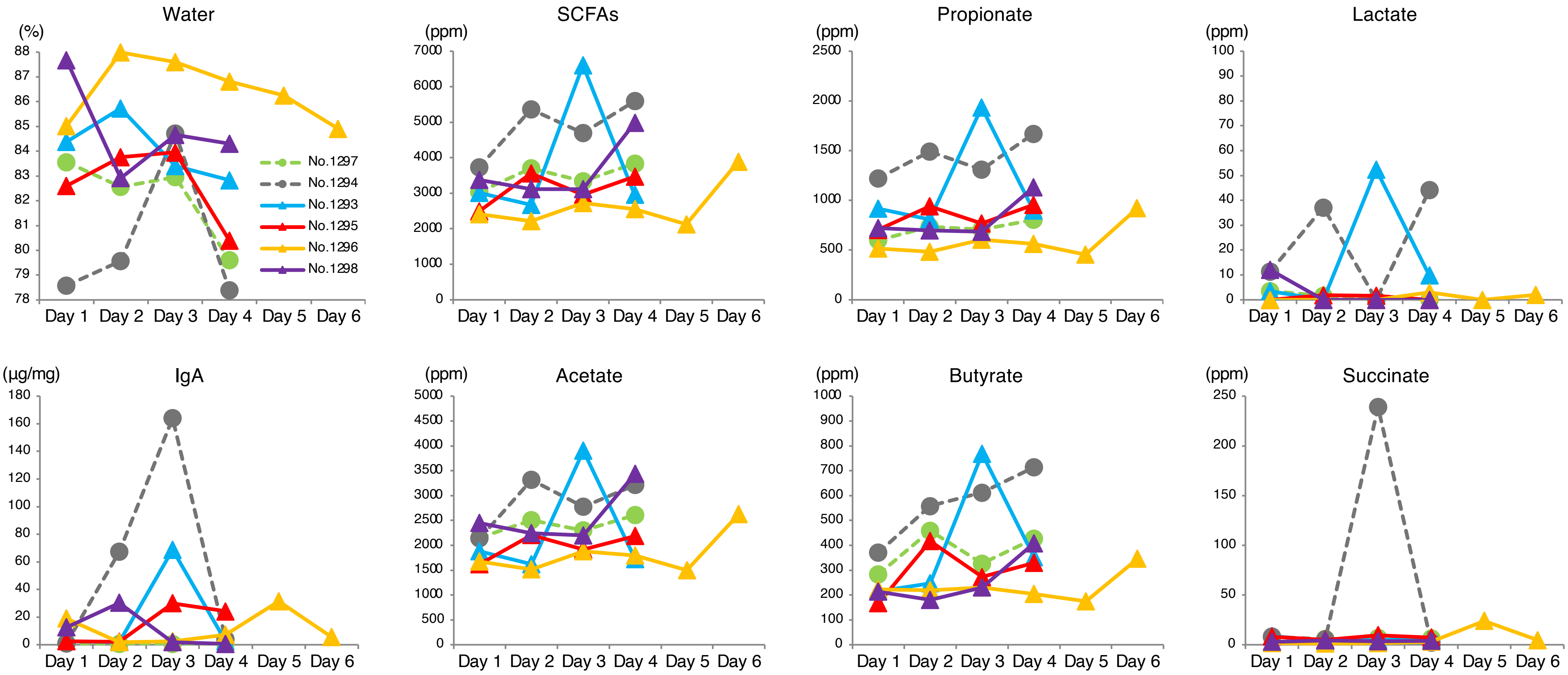

Fig.S2 Time course of faecal water content, IgA, SCFAs, lactate, and succinate in calves

The wavy line indicates data from calves that did not experience diarrhoea. Solid lines indicate data from calves in which diarrhoea was observed. Individual calf information is shown in Fig. S1a. The statistical values shown in Fig. 1b were calculated using three or four timepoints: the date of diarrhoea occurrence (Day 1, categorised as "Before" in Figs. S1a, d, and e), the following day (Day 2, categorised as "Next" in Figs. S1a, d, and e), the day immediately preceding the final timepoint (Day 2 or Day 4, categorised as "Semifinal" in Figs. S1a, d, and e), and the date of diarrhoea remission (Day 3 or Day 5, categorised as "Final" in Figs. S1a, d, and e).

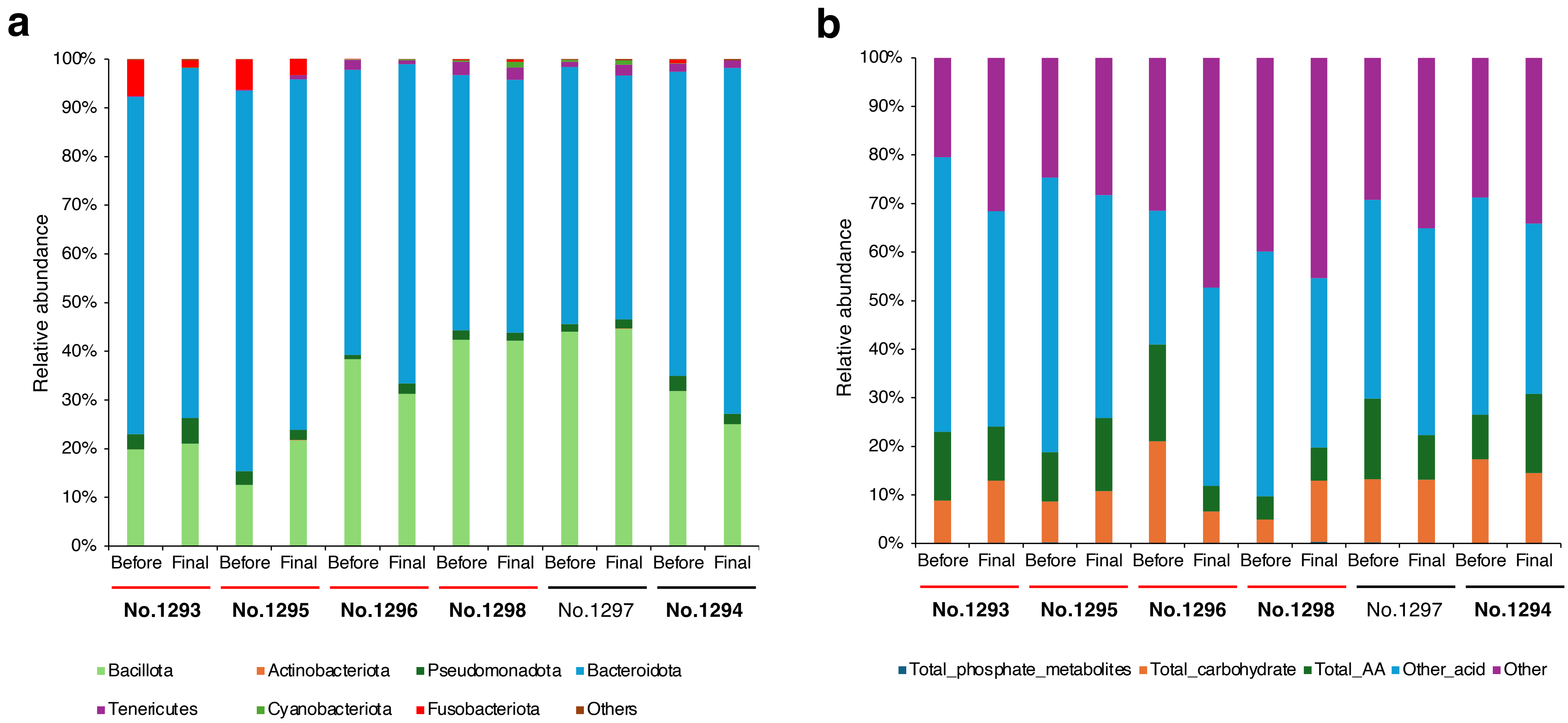

Fig.S3 Relative abundance of the faecal bacterial phyla and major metabolites in calves

The relative abundances of (**a**) phyla (1% or more as the maximum relative abundance) and (**b**) major metabolites in the faeces of cattle are visualized. The black line indicates an individual who has not developed diarrhoea, and the red line indicates an individual who has developed diarrhoea. “Before” and “Final” indicate Day 1 and Day 3 (or Day 5 in No. 1296) on the “Tested days” in Fig. S1A, respectively. The bold letters show the individuals administered the thermophile *Caldithermobacillus hisashii*, resulting in the five calves, except No. 1297, being administered the probiotic thermophile *C. hisashii* after the “Before” (Day 1).

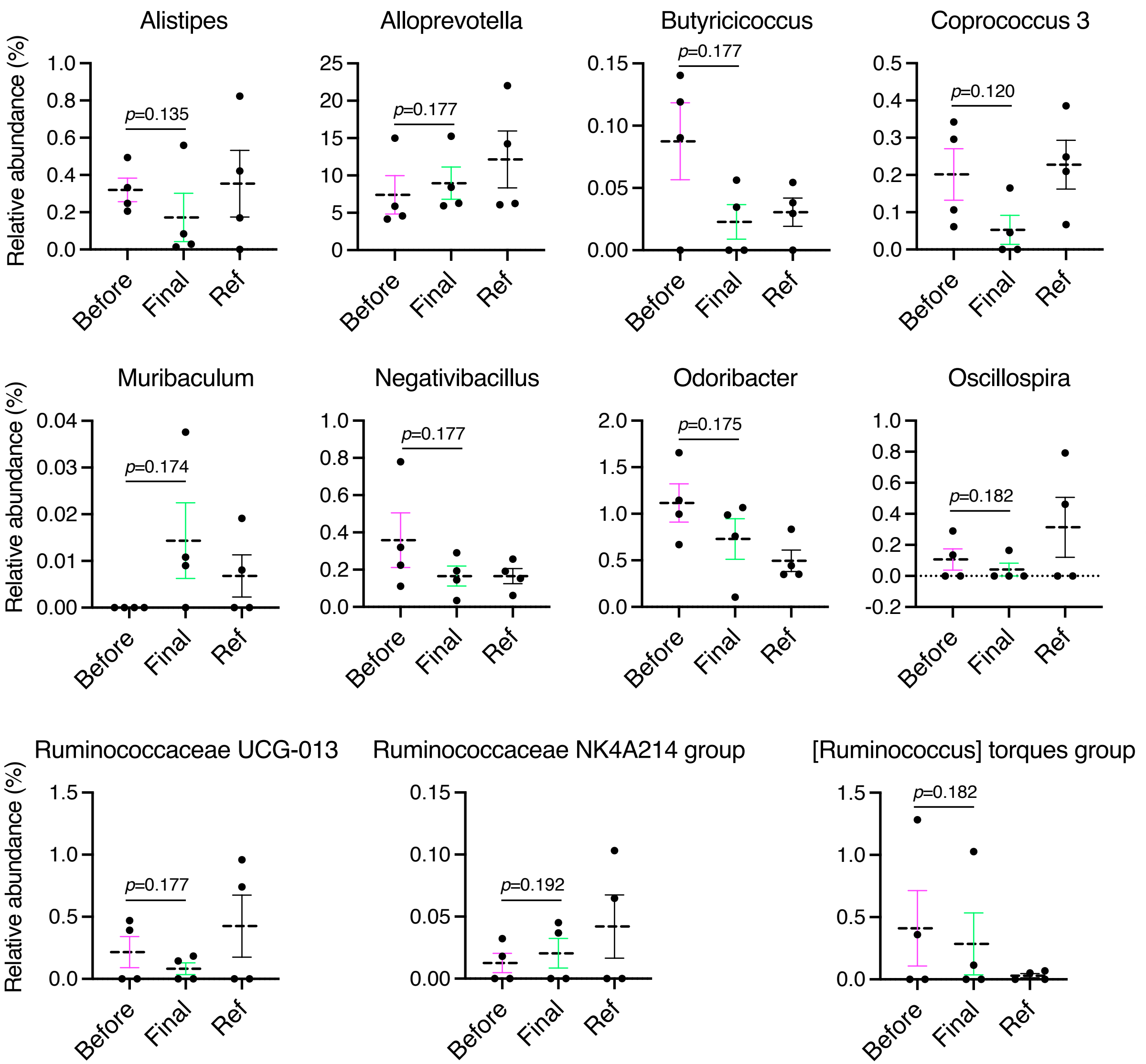

Fig.S4 Relative abundance of the faecal bacterial genera in calves

The relative abundances of bacterial genera in the faeces of calves are shown. "Before" indicates the observation day under diarrhoeal conditions, and "Final" indicates the day of recovery from diarrhoea. These correspond to Day 1 and Day 4 (or Day 6 for No. 1296) of the "Tested days" in Fig. S1A, respectively. Bold labels indicate individuals administered the probiotic thermophile *Caldithermobacillus hisashii*; five calves, excluding No. 1297, received *C. hisashii* after the "Before" time point (Day 1).

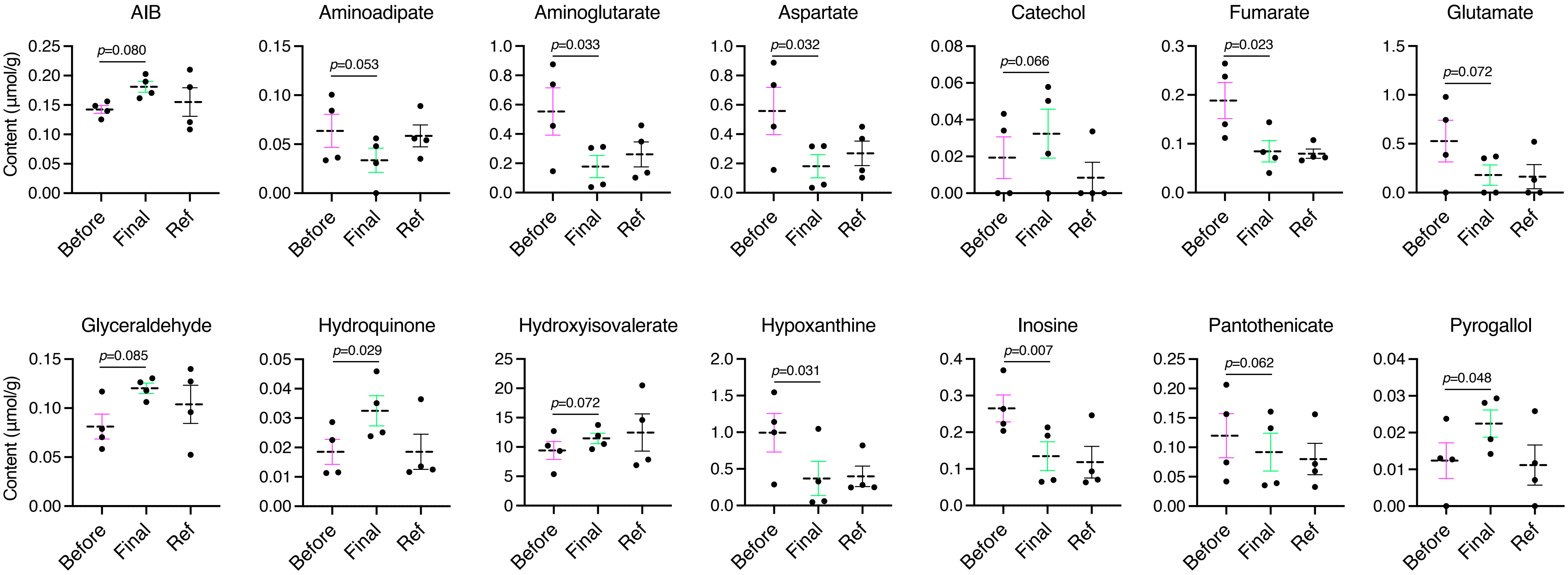

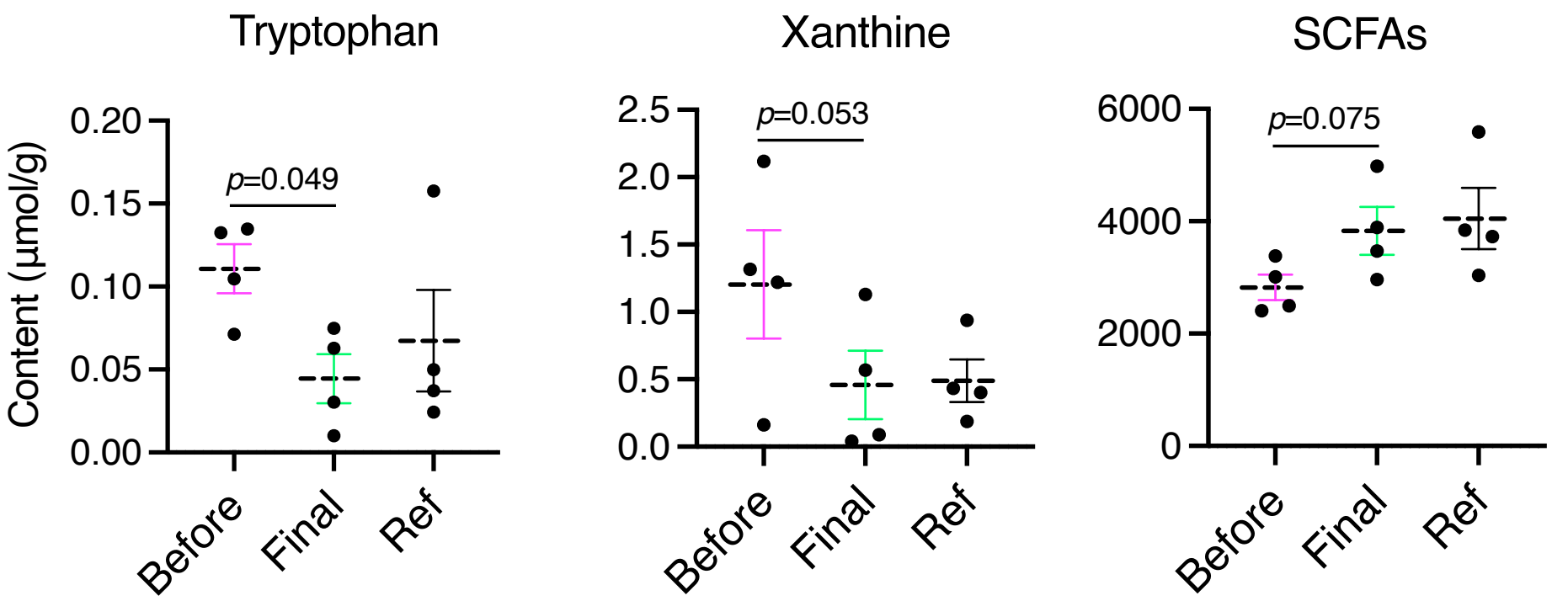

Fig.S5 Alterations in the faecal feature metabolites in calves

The levels of faecal metabolites are shown for each category. "Before" indicates the observation day under diarrhoeal conditions, and "Final" indicates the day of recovery from diarrhoea. These correspond to Day 1 and Day 4 (or Day 6 for No. 1296) of the "Tested days" in Fig. S1A, respectively. Bold labels indicate individuals administered the probiotic thermophile *Caldithermobacillus hisashii*; five calves, excluding No. 1297, received *C. hisashii* after the "Before" time point (Day 1).

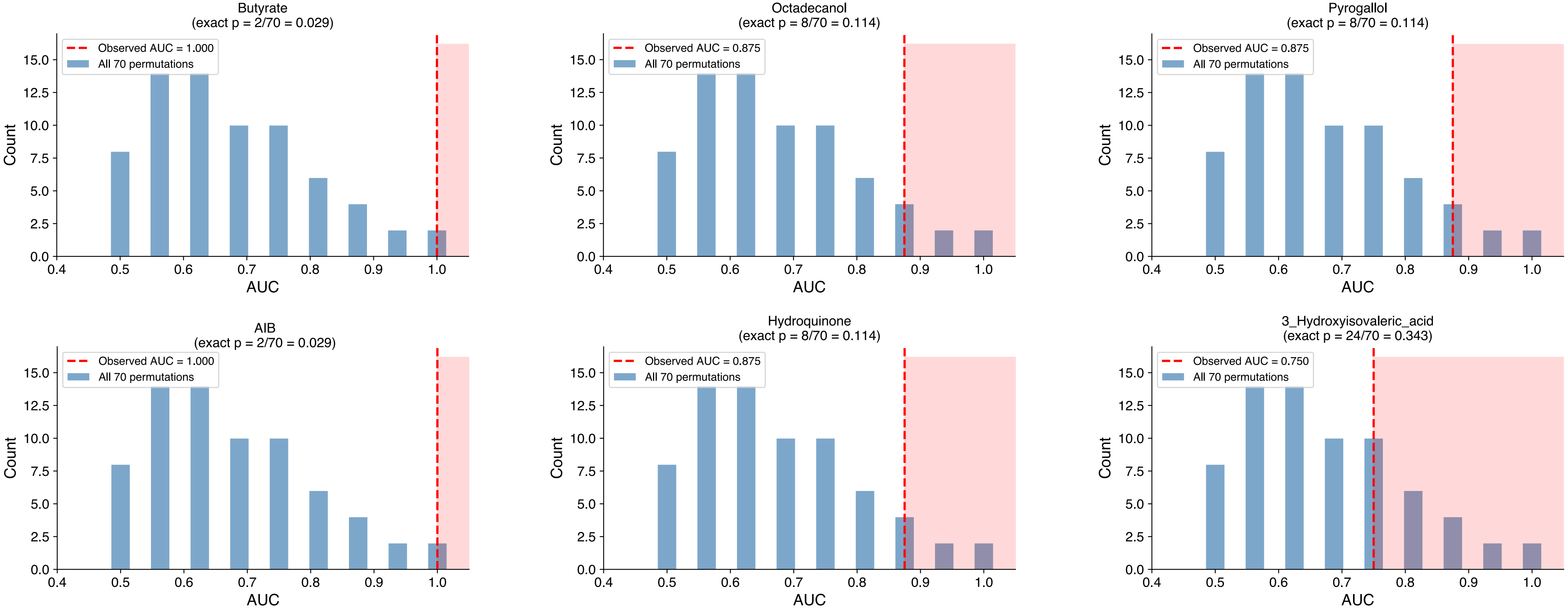

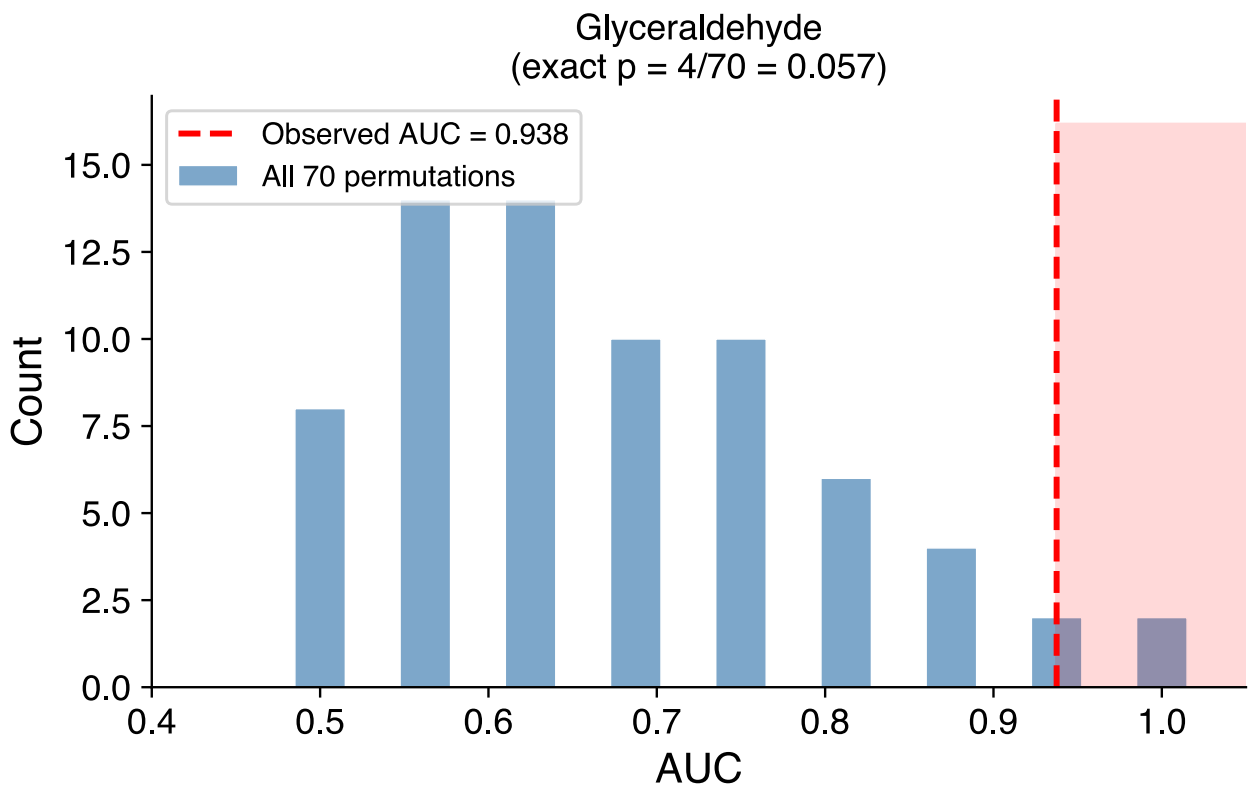

Fig.S6 Permutation tests for candidate feature metabolites with Cliff's delta = 1.0 in calves

Null distributions of AUC values (blue bars) were generated by randomly permuting group labels (Before/After) 70 times. Red dashed lines indicate the observed AUC for each metabolite. Pink shaded areas represent the proportion of permuted AUC values equal to or exceeding the observed AUC (i.e., the permutation p-value). Only butyrate and AIB achieved significant permutation *p*-values (p = 0.029), indicating that their complete group separation (AUC = 1.000) is unlikely to have arisen by chance. AUC, area under the receiver operating characteristic curve.

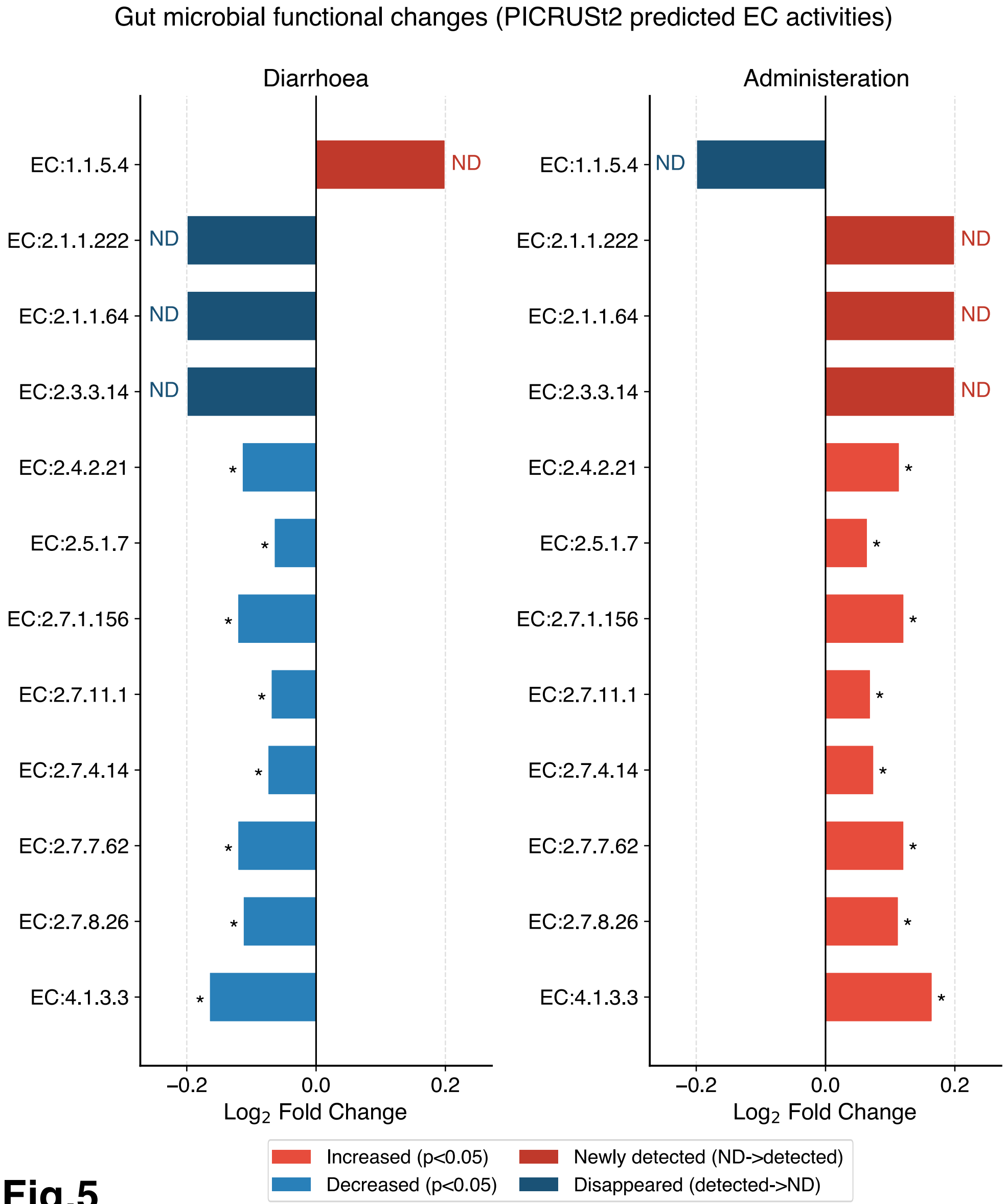

Fig.5

Fig.S7 Evaluation of gut bacterial metabolic function via the faecal 16S rRNA-based PICRUSt2 analysis in calves.

Log_2 fold changes in predicted enzymatic functions are shown for two comparisons: probiotic administration (left panel; administered vs non-administered calves) and diarrhoeal status (right panel; non-diarrhoeic vs diarrhoeic calves). Only EC numbers with $p < 0.05$ (*) (Mann-Whitney U test) are shown. CLR transformation was performed using Bayesian-multiplicative zero replacement (zCompositions) and the compositions R package. "ND" indicates that the function was detected in only one group (log_2FC not calculable). Given the small sample size (n = 4 per group), these results are presented as exploratory patterns. The EC numbers shown are: EC:1.1.5.4, malate dehydrogenase (quinone); EC:2.1.1.64, 3-demethylubiquinol 3-O-methyltransferase; EC:2.1.1.222, 2-polyprenyl- 6-hydroxyphenol methylase; EC:2.3.3.14, homocitrate synthase; EC:2.4.2.21, nicotinate-nucleotide- dimethylbenzimidazole phosphoribosyltransferase; EC:2.5.1.7, UDP-N-acetylglucosamine 1-carboxyvinyltransferase; EC:2.7.1.156, adenosylcobinamide kinase; EC:2.7.4.14, UMP-CMP kinase; EC:2.7.7.62, RNA 3'-terminal-phosphate cyclase; EC:2.7.8.26, phosphatidyltransferase; EC:2.7.11.1, non-specific serine/threonine protein kinase; and EC:4.1.3.3, N-acetylneuraminate lyase.

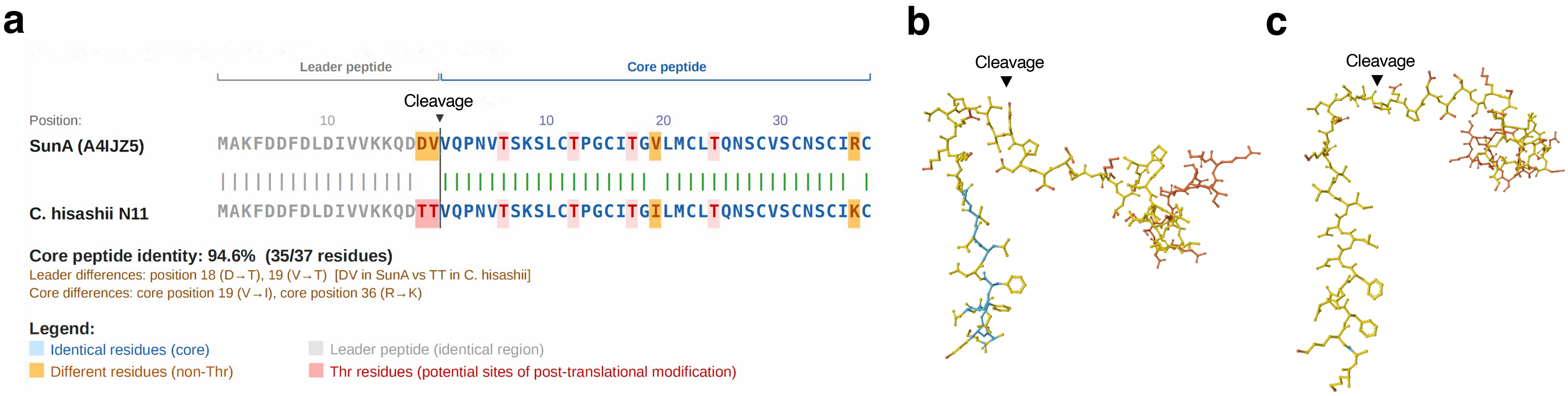

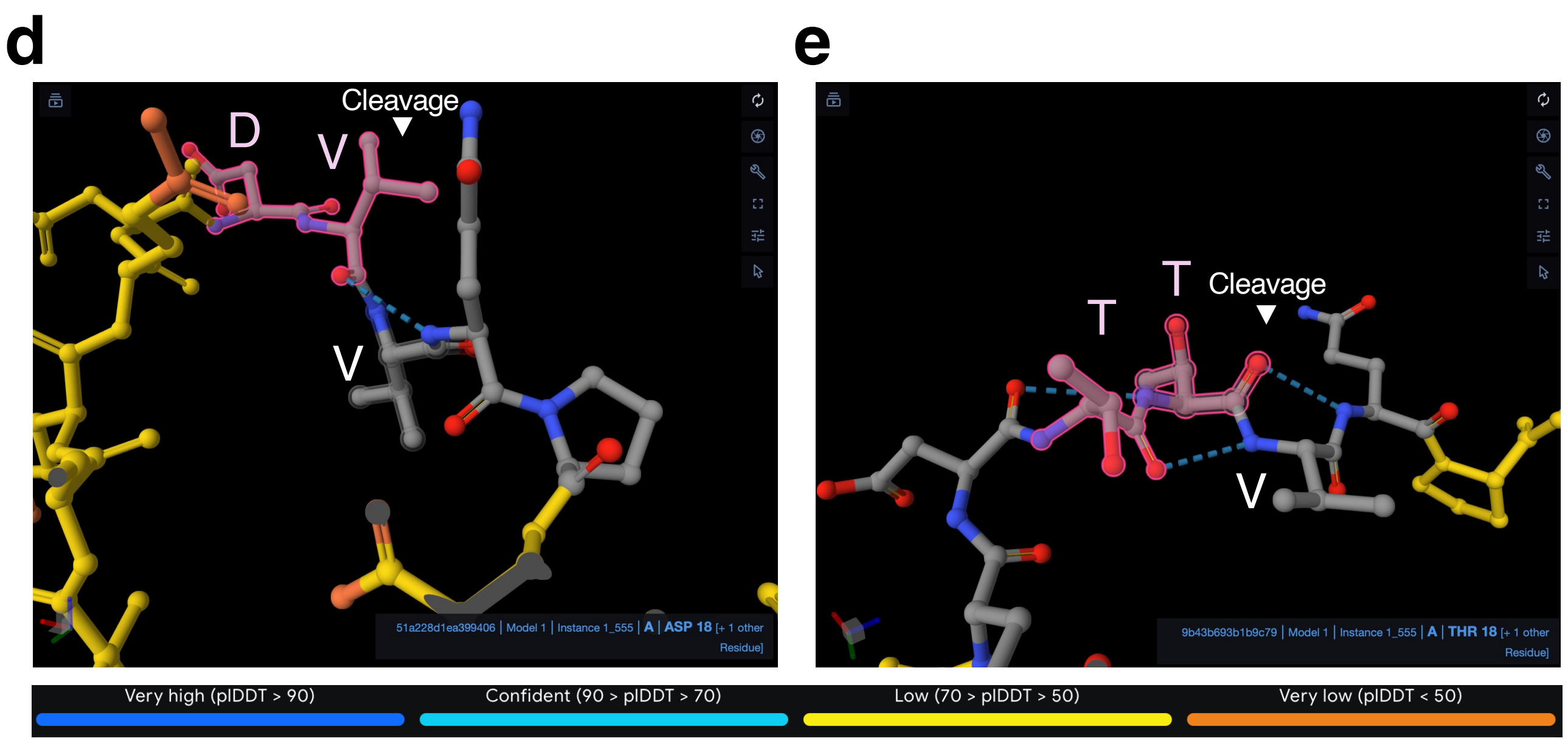

Fig.S8 Sequence alignment of precursor peptides of *Caldifermentibacillus hisashii* N11 and SunA (A4IJZ5)

(**a**) Pairwise alignment of the lanthipeptide core peptide precursor of *C. hisashii* N11 and SunA from *Bacillus subtilis* 168 (UniProt accession A4IJZ5; available at https://www.uniprot.org/uniprotkb/A4IJZ5/entry). Identical residues are shown with vertical bars indicating matches as legends. Orange highlighting indicates positions differing between the two sequences without Thr. Red letters with pink highlighting indicate Thr residues, which represent potential sites of post-translational modification by LanB dehydratase. Overall sequence identity is 92.9% (52 of 56 residues) and 94.6% as core peptide. Predicted structures of the unmodified precursor peptides generated by AlphaFold3 and coloured by pLDDT confidence score (blue, >90; cyan, 70–90; yellow, 50–70; orange, ≤50). Structures are displayed in ball-and-stick representation to show individual atomic details, in contrast to the ribbon representation used in Fig. 5. The cleavage site between leader and core peptide (▼) was shown in (**b**)(**d**) for SunA and (**c**)(**e**) for *C.hisashii* N11.

a

strain	Antibacterial activity by *C. hisashii*	
	Vegetative cells	Supernatant
Enterococcus gallinarum JCM8728	-	-
Escherichia coli K12	±	-
Staphylococcus aureus ATCC6538p	-	-
Streptococcus alactolyticus JCM31116	-	-
Streptococcus equinus JCM7879	-	-

Fig.S9 Antibacterial test of *Caldithermobacillus hisashii*
(**a**) Antibacterial activity against selected bacterial species. The symbols indicate the following: ±, antibacterial activity observed under certain conditions; –, antibacterial activity not confirmed. All cultures were incubated for up to 150 h. (**b**) Representative photographs of the antibacterial test against *Escherichia coli*. The abbreviations are as follows: Veg, vegetative cells; Sup, bacterial culture supernatant; and Amp, ampicillin (positive control).

b

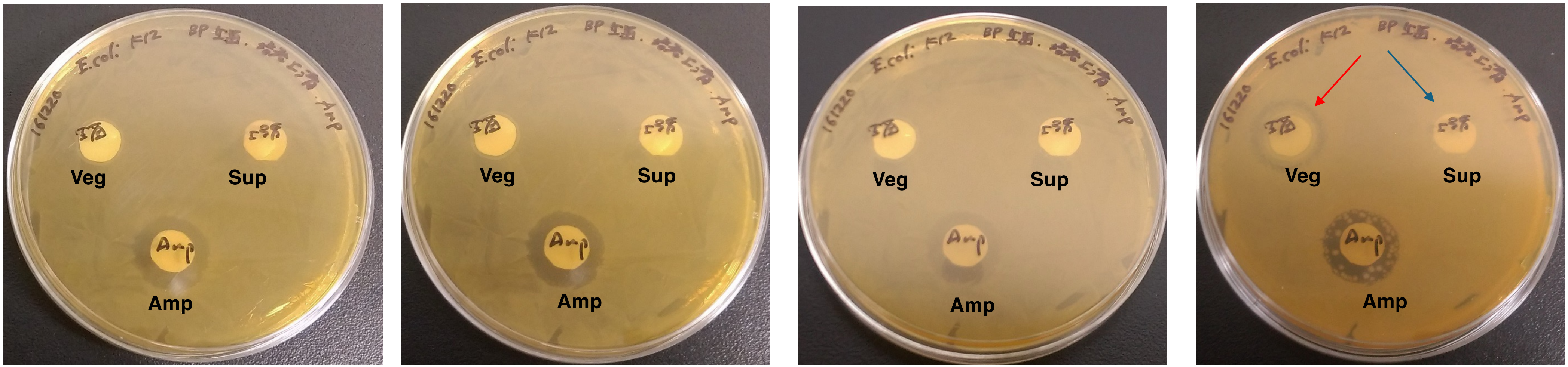

23h 47h 73h 143.5h

Table S1 Differentially predicted functions (EC numbers and pathways) identified by faecal 16S rRNA-based PICRUSt2 analysis in calves

EC numbers and pathways with $p < 0.05$ (Mann-Whitney U test) in the comparison between diarrhoeic and non-diarrhoeic states are shown. CLR diff indicates the difference in CLR-transformed mean abundance (non-diarrhoeic minus diarrhoeic). $\log_2$FC indicates $\log_2$(mean non-diarrhoeic / mean diarrhoeic); "Inf" and "-Inf" indicate that the function was detected in only one group. CLR transformation was performed using Bayesian-multiplicative zero replacement (zCompositions) and the compositions R package.

ID	Function	Direction	CLR diff	log2FC	p-value	Functional category
EC number						
EC:2.3.3.14	Homocitrate synthase	increase	4.090	Inf	0.029	Lysine biosynthesis
EC:1.1.5.4	Quinone-dependent malate dehydrogenase	decrease	-3.308	-Inf	0.029	TCA cycle / electron transport
EC:2.1.1.222	2-Polyprenyl-6-hydroxyphenol methylase	increase	1.014	Inf	0.029	Ubiquinone biosynthesis
EC:2.1.1.64	3-Demethylubiquinol 3-O-methyltransferase	increase	1.014	Inf	0.029	Ubiquinone biosynthesis
EC:4.1.3.3	N-acetylneuraminate lyase	increase	0.276	0.165	0.029	Amino sugar metabolism
EC:2.7.7.62	RNA 3'-terminal-phosphate cyclase	increase	0.260	0.121	0.029	RNA processing
EC:2.7.1.156	Adenosylcobinamide kinase	increase	0.260	0.121	0.029	Cobalamin biosynthesis
EC:2.4.2.21	Nicotinate-nucleotide-dimethylbenzimidazole phosphoribosyltransferase	increase	0.248	0.114	0.029	Cobalamin biosynthesis
EC:2.7.8.26	Phosphatidyltransferase	increase	0.245	0.113	0.029	Phospholipid biosynthesis
EC:2.7.11.1	Non-specific Ser/Thr protein kinase	increase	0.213	0.069	0.029	Signal transduction
EC:2.7.4.14	UMP-CMP kinase	increase	0.199	0.074	0.029	Nucleotide metabolism
EC:2.5.1.7	UDP-N-acetylglucosamine 1-carboxyvinyltransferase	increase	0.175	0.065	0.029	Peptidoglycan biosynthesis
Pathway						
PWY-7187	Pyrimidine deoxyribonucleotides de novo biosynthesis II	increase	0.211	0.126	0.029	Nucleotide biosynthesis

Table S2 Identification of genome-encoded genes involved in butyrate biosynthesis

Genes involved in butyrate biosynthesis were identified based on the complete genome sequence of *Caldifermentibacillus hisashii* N11 (AP028807.1; https://www.ncbi.nlm.nih.gov/nuccore/AP028807). Proteomic confirmation was performed using bacterial culture supernatant. The abbreviations are as follows: Abbr., abbreviation of protein name; Function, predicted biochemical function; Locus tag, genome locus identifier; Protein ID, DDBJ/GenBank protein accession number; Location (bp), chromosomal coordinates on AP028807.1; EC no., Enzyme Commission number; Identity (%), amino acid sequence identity to the registered genome sequence determined by tblastn analysis; Proteome, detection in supernatant proteome (Yes, confirmed; N.D., not detected); Figure, corresponding figure panel.

Protein name	Abbr.	Function	Locus tag	Protein ID	Location (bp)	EC no.	Identity † (%)	Proteome	Figure
**Butyrate biosynthetic gene cluster (2,843,472–2,848,237 bp, AP028807.1)**									
Acetyl-CoA C-acetyltransferase	AtoB	Butyrate biosynthesis (Step 1: acetyl-CoA → acetoacetyl-CoA)	CHN11BP863_26540	BES00121.1	2,843,472–2,844,656	EC:2.3.1.9	100	**Yes**	Fig. 5a
acyl-CoA dehydrogenase family protein	ACD	Butyrate biosynthesis (Step 4: crotonyl-CoA → n-butyryl-CoA)	CHN11BP863_26550	BES00122.1	2,844,665–2,845,819	–	100	**Yes**	Fig. 5a
enoyl-CoA hydratase-related protein	ECH	Butyrate biosynthesis (Step 3: 3-OH-butyryl-CoA → crotonyl-CoA)	CHN11BP863_26560	BES00123.1	2,845,850–2,846,644	EC:4.2.1.17	100	**Yes**	Fig. 5a
3-hydroxybutyryl-CoA dehydrogenase	BHBD	Butyrate biosynthesis (Step 2: acetoacetyl-CoA → 3-OH-butyryl-CoA)	CHN11BP863_26570	BES00124.1	2,846,631–2,847,476	EC:1.1.1.157	100	N.D.	Fig. 5a
CoA transferase subunit B	CtfB	Butyrate biosynthesis (Step 5: butyryl-CoA → butyrate)	CHN11BP863_26580	BES00125.1	2,847,590–2,848,237	–	99.5	N.D.	Fig. 5a
**Valine degradation – BKD complex (AP028807.1)**									
Lipoamide acyltransferase component of branched-chain alpha-keto acid dehydrogenase complex	bfmBB	Valine → isobutyryl-CoA (E2 subunit of BKD complex); substrate supply for IcmF	CHN11BP863_21770	BER99643.1	2,343,965–2,345,218	EC:2.3.1.168	100	**Yes**	–
Dihydrolipoyl dehydrogenase	lpdA_2	Valine → isobutyryl-CoA (E3 subunit of BKD complex); substrate supply for IcmF	CHN11BP863_21800	BER99646.1	2,347,317–2,348,735	EC:1.8.1.4	100	**Yes**	–

† Identity values of <100% reflect minor discrepancies between protein database sequences and tblastn translation of the registered genome (AP028807.1). All proteins were confirmed to be encoded in the registered genome of *Caldifermentibacillus hisashii* N11 (AP028807.1).

Table S3 Identification of genome-encoded genes involved in AIB-containing lantibiotic biosynthesis and self-immunity

Genes involved in AIB-containing lantibiotic biosynthesis, self-immunity, and AIB degradation were identified based on the complete genome sequence of *Caldifermentibacillus hisashii* N11 (AP028807.1; https://www.ncbi.nlm.nih.gov/nuccore/AP028807). Proteomic confirmation was performed using bacterial culture supernatant. The abbreviations are as follows: Abbr., abbreviation of protein name; Function, predicted biochemical function; Locus tag, genome locus identifier; Protein ID, DDBJ/GenBank protein accession number; Location (bp), chromosomal coordinates on AP028807.1; EC no., Enzyme Commission number; Identity† (%), amino acid sequence identity to the registered genome sequence determined by tblastn analysis; Proteome, detection in supernatant proteome (Yes, confirmed; N.D., not detected); Figure, corresponding figure panel.

Protein name	Abbr.	Function	Locus tag	Protein ID	Location (bp)	EC no.	Identity† (%)	Proteome	Figure
**Lanthipeptide BGC – Region 2 (3,744,454–3,770,832 bp, AP028807.1)**									
NisI/SpaI family lantibiotic immunity lipoprotein	NisI	Self-immunity: lantibiotic binding/neutralization (lipoprotein)	CHN11BP863_34730	BES00940.1	3,746,710–3,747,156	—	99.1	N.D.	Fig. 5b
Lantibiotic immunity ABC transporter permease (MutG family)	MutG	Self-immunity: protection against lantibiotic (permease subunit)	CHN11BP863_34750	BES00942.1	3,747,192–3,747,980	—	100	N.D.	Fig. 5b
Lantibiotic immunity ABC transporter permease (MutE/EpiE family)	MutE	Self-immunity: protection against lantibiotic (permease subunit)	CHN11BP863_34760	BES00943.1	3,747,982–3,748,728	—	100	N.D.	Fig. 5b
Lantibiotic protection ABC transporter ATP-binding protein	LanF	Self-immunity: protection against lantibiotic (ATPase subunit)	CHN11BP863_34770	BES00944.1	3,748,744–3,749,436	—	100	N.D.	Fig. 5b
Lanthipeptide cyclase	LanC	Thioether ring formation (lanthionine/methyllanthionine bridges)	CHN11BP863_34820	BES00949.1	3,754,454–3,755,761	—	100	N.D.	Fig. 5b
Lantibiotic ABC transporter (SrtF homolog)	LanT	Lantibiotic secretion across cell membrane	CHN11BP863_34830	BES00950.1	3,755,730–3,757,562	—	100	**Yes**	Fig. 5b
Lanthipeptide dehydratase	LanB	Lanthipeptide dehydration (Ser/Thr → Dha/Dhb)	CHN11BP863_34840	BES00951.1	3,757,692–3,760,832	—	100	N.D.	Fig. 5b
Core peptide precursor (gallidermin/nisin family lantibiotic)	LanA	AIB-containing lantibiotic precursor (56 AA; 7 Cys residues)	CHN11BP863_34850	BES00952.1	3,761,380–3,761,550	—	100	N.D.	Fig. 5b
IcmF (annotated as methylmalonyl-CoA mutase family protein)	IcmF	Isobutyryl-CoA ⇌ n-butyryl-CoA (metabolic link: Butyrate and AIB); 79.2% identity to G. kaustophilus IcmF (Q5KUG0)	CHN11BP863_34890	BES00956.1	3,765,994–3,769,269	EC:5.4.99.13	100	**Yes**	Fig. 5b
TetR/AcrR family transcriptional regulator	TetR	Transcriptional regulation of lantibiotic biosynthetic gene cluster	TBD	TBD	3,769,418–3,770,080				
**BGC-distal protein (AP028807.1)**									
DUF1796 family putative cysteine peptidase (putative papain-like cysteine peptidase)	—	Putative leader peptide cleavage (located 1.74 Mb upstream of Region 2)	CHN11BP863_18310	BER99297.1	2,003,731–2,004,360	—	100	**Yes**	Fig. 5c
**AIB metabolism (AP028807.1)**									
alanine–glyoxylate aminotransferase family protein	D-BAIBAT	D-β-aminoisobutyrate (BAIBA) degradation: D-β-AIB → methylmalonate semialdehyde → propionyl-CoA	CHN11BP863_03220	BER97788.1	355,757–356,938	EC:2.6.1.44	100	**Yes**	—

† Identity values of <100% reflect minor discrepancies between protein database sequences and tblastn translation of the registered genome (AP028807.1). All proteins were confirmed to be encoded in the registered genome of *Caldifermentibacillus hisashii* N11 (AP028807.1).

Table S4 Identification of biosynthetic gene clusters (BGCs) in *Caldifermentibacillus hisashii* N11

BGCs were predicted using antiSMASH (v8.0.4) based on the complete genome sequence of *Caldifermentibacillus hisashii* N11 (AP028807.1; https://www.ncbi.nlm.nih.gov/nuccore/AP028807). The abbreviations are as follows: Type, predicted BGC type based on antiSMASH classification; Location (nt), chromosomal coordinates of the BGC on AP028807.1; Total (nt), total length of the BGC in nucleotides; Core, number of core biosynthetic genes; Additional, number of additional biosynthetic genes; Transport, number of transport-related genes; Regulatory, number of regulatory genes; Other, number of other genes within the BGC; and Note, additional remarks including proteomic evidence and reference to corresponding figures.

**antiSMASH Region no.**	**1**	**2**	**3**
Type	Terpene precursor	Class I Lanthipeptide	Betalactone
Location (nt)	2,351,069 - 2,371,950	3,744,454-3,770,832	3,841,323 - 3,865,649
total (nt)	20,882	26,379	24,327
Core	1	2	2
Additional	2	1	7
Transport	0	2	1
Regulatory	1	4	2
Other	18	9	7
Note	Precursor only	See Fig. 6c	HMGL-like + AMP-binding
